# Multi‐Parameter Spectral Flow Cytometry Panel for Immune Phenotyping of Murine B and T Cell Responses

**DOI:** 10.1002/eji.70182

**Published:** 2026-04-02

**Authors:** Kassandra Hoetzel, Hendrik Feuerstein, Julia Ludwig, Hedda Wardemann

**Affiliations:** ^1^ B Cell Immunology German Cancer Research Center (DKFZ) Heidelberg Germany; ^2^ Faculty of Biosciences Heidelberg University Heidelberg Germany

**Keywords:** B cells, spectral flow cytometry, T cells, vaccination

## Abstract

To enable the analysis of B cell and T cell immune responses in mouse lymph nodes, spleen, and bone marrow, including different B cell and T cell subsets and their activation status, as well as the antigen‐reactivity and isotype of B cells, a novel 31‐parameter spectral flow cytometry panel was developed.

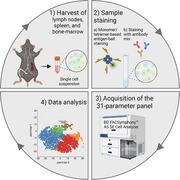

**Abbreviations**
CM: Central memory
EM: Effector memory
FO: Follicular
GC: Germinal center
MZ: Marginal zone
PaCMAP: Pairwise controlled manifold approximation and projection
PB: Plasmablast
PC: Plasma cell
RBD: SARS‐CoV‐2 receptor‐binding domain
S6P: SARS‐CoV‐2 prefusion spike protein stabilized by six prolines
TACI: Transmembrane activator and CAML interactor
Tfh: T follicular helper (cell)

The primary site of T cell‐dependent B cell immune responses are the germinal centers (GCs) within secondary lymphoid organs [1]. In these microanatomical structures, antigen‐activated B cells undergo affinity maturation, a process in which somatically mutated B cells with improved antigen‐binding capacity are selected through competition for antigen and T cell help, before differentiating into antibody‐secreting cells and memory B cells [1, 2]. While the majority of newly generated antibody‐secreting cells are short‐lived plasmablasts (PB), a subset can home to dedicated survival niches in the bone marrow and differentiate into long‐lived plasma cells (PCs), thereby mediating durable humoral immunity. During recall responses, memory B cells rapidly develop into antibody‐secreting cells, contributing to the swift production of antigen‐specific serum antibodies.

Here, we describe a 31‐parameter spectral flow panel for the deep phenotypic analysis of antigen‐specific B cell responses [3, 4, 5, 6, 7, 8, 9, 10, 11, 12, 13, 14]. The panel was established using a full visible spectrum cytometer (BD FACSymphony A5 SE Cell Analyzer) equipped with 5 lasers and 49 detectors (Tables S1 and S2, Figure S1) and lymph node, spleen, and bone marrow samples from non‐immunized mice and mice immunized with trimeric SARS‐CoV‐2 prefusion spike protein stabilized by six prolines (S6P). Antigen‐reactive B cells were identified using fluorochrome‐labeled S6P and SARS‐CoV‐2 receptor‐binding domain (RBD) baits. The materials and methods, including the antibody list (Table S3), single‐color reference controls and N × N plots (Table S4, Figure S2), the antibody titration process (Figure S3), and antigen bait staining (Figures S4 and S5) as well as limitations of the study are described in the Supporting Information. Moreover, a summary of the panel design and optimization process is provided in Tables S5 and S6.

Regardless of the tissue of origin, single lymphocytes from all organs were identified based on their size and granularity (Figures 1A and  2A). Next, dead cells, as well as natural killer cells, erythrocytes, monocytes (including neutrophils), macrophages, and dendritic cells (collectively referred to as lineage‐negative [Lin^−^] cells), were excluded from the analysis. This was achieved using a fixable viability stain (440UV) and a lineage antibody mix (anti‐NK‐1.1, anti‐TER‐119, anti‐Ly‐6G/Ly‐6C [Gr‐1], anti‐F4/80, and anti‐CD11c), all conjugated to the same fluorochrome [3]. Then, sequential gating was applied to identify each B and T cell subset unambiguously.

**FIGURE 1:**
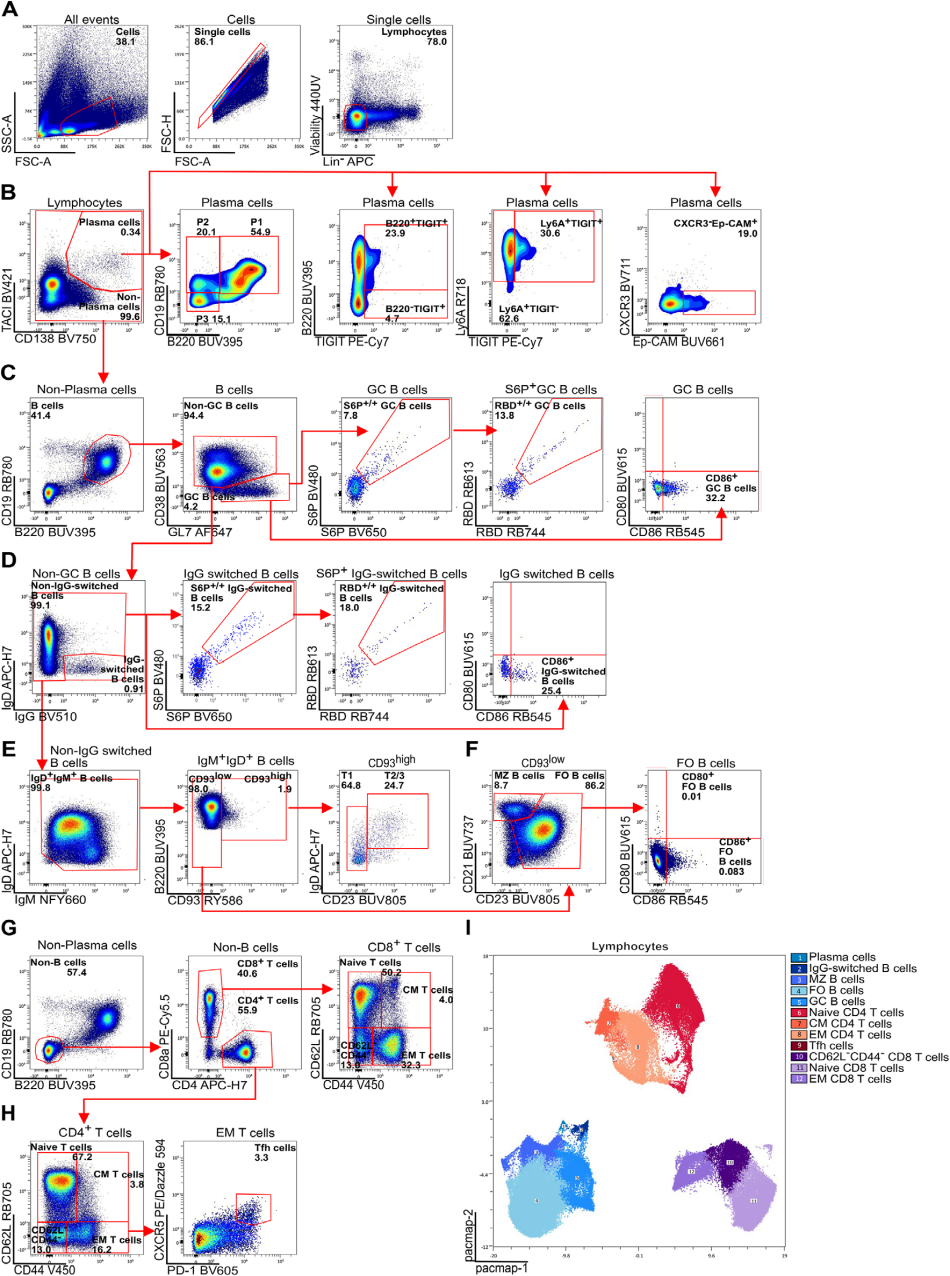
Gating strategy for murine spleen samples from an immunized mouse. (A) Viable lymphocytes. (B) PCs (TACI^int^CD138^+^) were further subdivided into three subsets: P1 (B220^int^CD19^int^), P2 (B220^low^CD19^int^), and P3 (B220^low^CD19^low^). PCs with Ep‐CAM^high^CXCR3^−^ or Ly6A^high^TIGIT^−^ phenotypes were identified. (C) GC B cells (CD38^−^GL7^+^) were investigated for antigen reactivity (S6P^+^^/^^+^, RBD^+^^/^^+^) and activation status (CD86^+^). (D) IgG^+^ class‐switched B cells (CD38^+^GL7^−^IgG^+^) showed antigen‐reactivity (S6P^+^^/^^+^, RBD^+^^/^^+^) and expressed the activation marker CD86. (E) B220^+^CD93^high^ T1‐ (IgM^high^CD23^low^) and T2‐stage (IgD^high^CD23^high^) transitional B cells were identified. (F) B220^+^CD93^low^ naive follicular (CD23^high^CD21^+^) and marginal zone (CD23^low^CD21^high^) B cells were defined. Activated naive follicular B cells (CD80^+^ or CD86^+^) were characterized. (G) Among CD8^+^ T cells, naive (CD44^−^CD62L^+^), CM (CD44^+^CD62L^+^), and EM (CD44^+^CD62L^−^) were distinguished. (H) Similarly, naive, CM, and EM CD4^+^ T cells were distinguished. EM CD4^+^ T cells were further gated to identify Tfh cells (CXCR5^+^PD‐1^+^). (I) PaCMAP (2D; kNN = 30, mid = 0.5, far = 2600 iters, Euclidean, random init) with FlowSOM metaclusters (*k* = 16) overlaid to show B/T‐cell phenotypic heterogeneity. Data shown are representative of three mice (*n* = 3) from one experiment.

**FIGURE 2:**
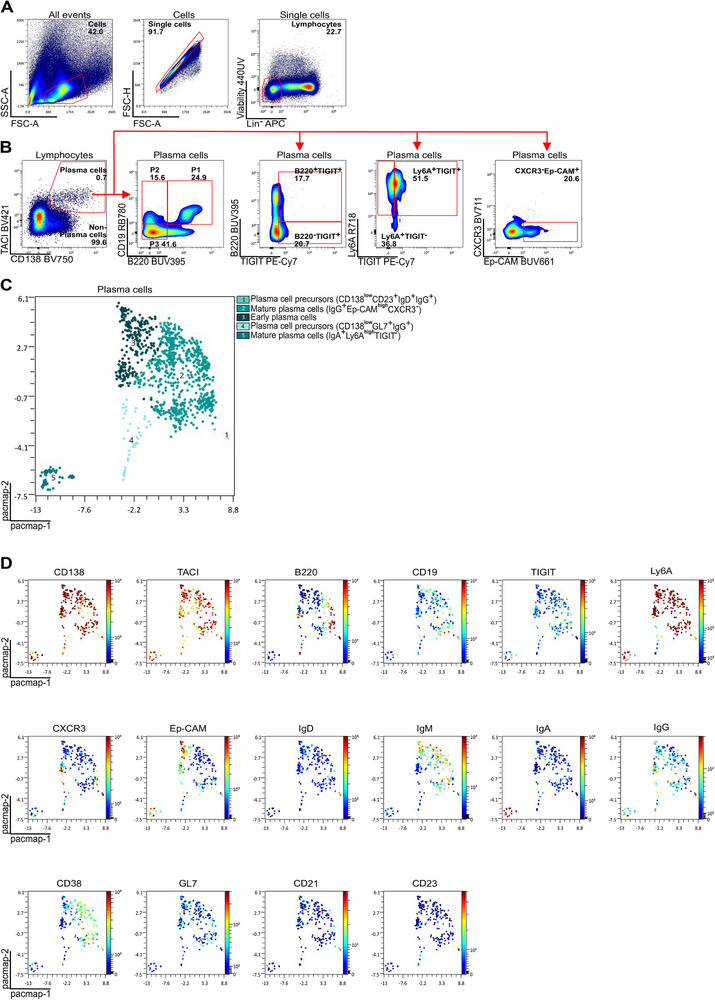
Gating strategy for murine bone marrow PC subsets. (A) Viable lymphocytes. (B) PCs (TACI^int^CD138^+^) subsets: P1 (B220^int^CD19^int^), P2 (B220^low^CD19^int^), and P3 (B220^low^CD19^low^). PCs with Ep‐CAM^+^CXCR3^−^ or Ly6A^+^TIGIT^−^ phenotypes were distinguished. (C) PaCMAP (2D; kNN = 30, mid = 0.5, far = 2600 iters, Euclidean, random init, seed = 4786) with FlowSOM metaclusters (*k* = 5) overlaid to show antibody‐secreting cell differentiation diversity. (D) Selected marker expression is overlaid as a continuous color gradient on the PaCMAP to define PC subsets. Data shown are representative of three mice (*n* = 3) from one experiment.

Immunization led to the development of PCs, which were identified by the expression of CD138 and the transmembrane activator and CAML interactor (TACI) (Figures 1B and 2B, Figures S6, S9, S10, and S11) [11, 12, 13]. Three PC subsets (P1‐3) were discriminated based on their CD19 and B220 expression: P1‐ PC precursors (B220^int^CD19^int^), P2‐early PCs (B220^low^CD19^int^), and P3‐mature PCs (B220^low^CD19^low^). The panel includes markers tailored for the analysis of long‐lived PCs [12] including antibodies against TIGIT, which is predominantly expressed on PC precursors (P1) and early PCs (P2), as well as CXCR3, Ep‐CAM, and Ly6A, markers associated with IgG (Ep‐CAM**^+^** CXCR3**^−^**) or IgA (Ly6A**^+^** TIGIT**^−^**) expression [12, 13].

B cells were identified based on the lineage markers CD19 and B220. Among these, GC B cells were defined as GL7^+^CD38^−^. While GC B cells were rare in non‐immunized mice, they were clearly detectable in the spleen and lymph nodes of immunized animals (Figure 1C and Figures S6, S9, and S10). These GC B cell populations included a substantial fraction of S6P and RBD bait‐reactive cells, as well as CD86^high^ cells, phenotypically resembling light zone GC B cells (Figure 1C) [1, 7, 8, 9, 10].

In the spleen, CD38 was expressed on marginal zone (MZ), naive follicular (FO), transitional, and IgG^+^ class‐switched B cells (Figure S7). To better separate the IgG^+^ class‐switched B cells, isotype‐specific antibodies were used (Figure S8). The frequency of IgG^+^ class‐switched B cells was higher in immunized mice, including IgG^+^ cells that bound antigen (S6P, RBD) and expressed the activation marker CD86 reminiscent of memory B cells (Figure 1D and Figure S6). Antibodies against CD93, CD21, and CD23 enabled the discrimination of transitional B cell subsets (T1 – CD93^high^IgD^neg/low^CD23**^−^**; T2/T3 – CD93^low^IgD^low/pos^CD23^+^) from MZ (IgM^+^CD21^high^CD23^low^) and naive FO (IgD^+^IgM^int‐low^CD23^high^CD21^+^) B cells in spleen (Figure 1E,F) [4, 5, 8]. In immunized, but not in non‐immunized, mice, naive FO B cells expressed higher levels of the activation marker CD80 but not CD86 (Figure 1F and Figure S6).

To facilitate the parallel assessment of T cell responses, antibodies against CD4 and CD8 were included in the panel (Figure 1G) [1, 8, 14, 15]. CD4 and CD8 T cells were further characterized based on the expression of CD44 and CD62L to distinguish naive (CD44**^−^** CD62L^+^), effector memory (EM; CD44^+^CD62L**^−^**), and central memory (CM; CD44^+^CD62L^+^) subsets (Figure 1G,H). CXCR5 and PD‐1 expression on EM CD4 T cells was used to identify T follicular helper (Tfh) cells (Figure 1H). As CD62L is sensitive to enzymatic shedding, we caution that its detection may be impaired in thawed samples due to ex vivo processing, affecting the detection of naive and CM T cell subsets. To preserve reliable CD62L staining, we recommend using freshly prepared samples, minimizing incubation steps at room temperature, avoiding mechanical stress, and keeping samples on ice during processing whenever possible (Figure S12). Pairwise controlled manifold approximation and projection was used to distinguish the diverse cellular subsets (Figures 1I and 2C,D, Figure S13).

In summary, this 31‐parameter spectral flow cytometry panel allows for the high‐resolution phenotypic discrimination of diverse murine B and T cell subsets, with particular focus on antigen‐experienced B cells, including rare subpopulations such as long‐lived PCs and isotype‐switched (memory) B cells. It is ideally suited for analyzing immunization or infection‐induced immune responses in lymph nodes, spleen, and bone marrow.

## Author Contributions

H.W. designed the study, interpreted data, and wrote the manuscript. K.H. designed the study, performed experiments, interpreted data, and wrote the manuscript. H.F. and J.L. performed experiments and interpreted data.

## Funding

This work was supported by the Helmholtz Association's Initiative and Networking Fund project “Virological and immunological determinants of COVID‐19 pathogenesis‐lessons to get prepared for future pandemics (KA1‐Co‐02_CoViPa).” H.F. and K.H. were supported by the DKFZ International PhD Program.

## Ethics Statement

All C57BL/6J mouse procedures were approved by the Regierungspräsidium Karlsruhe, Germany (Project Number: G27/23) and experiments were conducted in accordance with the German Animal Protection Law.

## Conflicts of Interest

The authors declare no conflicts of interest.

## Supporting information




**Supporting File**: eji70182‐sup‐0001‐SuppMat.pdf.

## Data Availability

The flow cytometry files are available from the corresponding author upon request.
