# Supplementary material for: Multi‐Parameter Spectral Flow Cytometry Panel for Immune Phenotyping of Murine B and T Cell Responses
Source: Eur J Immunol. 2026 Apr 2;56(4):e70182. doi: 10.1002/eji.70182 (PMC13047354; doi:10.1002/eji.70182)
Supplement: Supplementary file 1 — Supporting File: eji70182‐sup‐0001‐SuppMat.pdf. [file EJI-56-e70182-s001.pdf]

Supplementary Information

Multi-parameter spectral flow cytometry panel for immune phenotyping of murine B and T cell responses

Kassandra Hoetzel[1,2] (ORCID ID: 0009-0009-6076-7602, kassandra.hoetzel@dkfz-heidelberg.de), Hendrik Feuerstein[1,2] (ORCID ID: 0000-0002-8698-1844, hendrik.feuerstein@dkfz-heidelberg.de), Julia Ludwig[1] (ORCID ID: 0009-0005-2624-8180, j.ludwig@dkfz-heidelberg.de), Hedda Wardemann[1] (ORCID: 0000-0003-3921-5933, h.wardemann@dkfz-heidelberg.de)

[1] B Cell Immunology, German Cancer Research Center (DKFZ), Heidelberg, Germany

[2] Faculty of Biosciences, Heidelberg University, Heidelberg, Germany

Data Limitations and Perspectives

While our panel is optimized for the identification of major B and T cell subsets in the context of immunization and infection, certain limitations should be considered when applying it to broader immunophenotyping studies. The inclusion of CD11c and Gr-1 in the lineage exclusion mix may impact the detection of specific populations: CD11c is known to be expressed on atypical or age-associated B cells, as well as activated B cell subsets, and Gr-1 (Ly6G/Ly6C) may bind to Ly6C expressed on Th1 $CD4^+$ T cells. Researchers aiming to characterize these populations in detail should consider modifying the dump channel or applying more nuanced gating strategies.

In addition, CD62L expression is highly sensitive to ex vivo handling and may be reduced due to enzymatic shedding during thawing or extended incubation at room temperature. This can affect the resolution of naive and central memory T cells. For accurate detection of CD62L, we recommend using freshly prepared samples, minimizing room temperature processing, and keeping cells on ice during staining whenever possible.

While fluorescence minus one (FMO) controls are valuable for setting gates in conventional flow cytometry, the application of spectral flow cytometry panels with a large number of parameters presents specific challenges. In particular, limited sample availability and the need to stain for numerous markers within a single panel often limit the feasibility of generating FMOs for every marker. In such cases, single-stained reference controls and unstained samples become essential for panel validation and data interpretation. To address this, we provided extended control analyses in the supplementary material (Figs. S6, S10, S11), and assessed unmixing accuracy using NxN plots where each marker is plotted against all others (Fig. S2). These plots offer a comprehensive overview of population separation, signal spread, and potential issues with autofluorescence or spectral overlap. While FMOs remain a useful tool in certain applications, we recommend that users evaluate their utility based on the specific design and constraints of their experiments. These considerations highlight the importance of adapting marker panels and workflows to the specific biological questions and technical constraints of a given study.

Finally, it is important to note that surface marker expression provides only an indirect approximation of functional cell states such as activation, exhaustion, or effector potential. While our panel includes markers commonly associated with these functional attributes, definitive conclusions about cellular function cannot be drawn without complementary assays such as cytokine production, proliferation, or cytotoxicity measurements. We therefore recommend that researchers interested in confirming functional phenotypes consider integrating additional experimental approaches alongside this phenotyping panel.

Supplementary Materials and Methods:

Description of the materials and methods used to prepare single cell suspensions from lymph nodes, spleen, and bone marrow.

Material:

Commercially available reagents

- PBS (Gibco, Cat. 70011-036)
- Fetal bovine serum (FBS) (Sigma-Aldrich, Cat. TMS-013)
- Dimethylsulfoxid (Sigma-Aldrich, Cat. D2650)
- 40 µM nylon cell strainer (Corning, Cat. 431750)
- Cryogenic vial (Corning, Cat. 431417)
- Corning® CoolCell™ FTS30 (Corning, Cat. 432006)
- BD Brilliant Buffer (BD Biosciences, Cat. 566349)
- RPMI 1640 (Gibco, Cat. 21875034)
- Dulbecco's Phosphate Buffered Saline (DPBS) (Gibco, Cat. 14190-144)
- Neubauer counting chamber (Millipore, Cat. MDH-2N1-50PK)
- Trypan blue stain 0.4% (Life Technologies, Cat. T10282)
- Fixable Viability Stain 440UV (BD Biosciences, Cat. 566332)
- Fc-Block – purified anti-mouse CD16/32 antibody (Invitrogen, Cat. 14-0161-82)
- CellBlox™ blocking buffer (Thermo Fisher Scientific, Cat. B001T06F01)

Buffers

<u>Freezing medium</u>: 80% (v/v) Fetal bovine serum, 20% (v/v) Dimethylsulfoxid

<u>Staining buffer</u>: 2% (v/v) Fetal bovine serum in PBS

<u>Live-Dead dye solution</u>: Dilute fixable Viability Stain 440UV 1:500 in Dulbecco's Phosphate Buffered Saline

<u>Brilliant staining buffer</u>: Mix 1 part of BD Brilliant Buffer with 1.8 parts of Staining buffer

Methods:

Mice (C57BL/6J, aged 6–8 weeks) received two intramuscular immunizations, four weeks apart, with SARS-CoV-2 prefusion spike protein stabilized by six prolines (S6P) formulated in liposome-based adjuvant (LMQ); or were left non-immunized as controls. Mice were sacrificed one week after the second immunization, and samples were processed and cryopreserved as described below.

Lymph node and spleen processing protocol:

1. Dissected inguinal, axial, and popliteal lymph nodes or spleen from mice and transferred them into falcons with PBS.

2. Pressed tissue pieces through a 40 µm cell strainer into the same falcon filled with PBS using a syringe plunger.
3. Centrifuged at 403 × g for 10 minutes at 4°C.
4. Removed the supernatant, resuspended lymph node cells in 400 µl FBS, counted cells, and added 400 µl freezing medium (3 million cells per tube).
   Removed the supernatant, resuspended splenocytes in 900 µl FBS, counted cells, and added 900 µl freezing medium (6 million cells per tube).
5. Froze cells immediately in a Corning® CoolCell™ FTS30 at -80°C overnight.
6. Transferred cells to liquid nitrogen the next day.

Bone marrow processing protocol:

1. Prepared tibia and femur from mice with scissors and cut the bones open at the ends.
2. Placed the bones into an Eppendorf tube and centrifuged at 956 × g for 6 minutes at 4°C to flush out the cells.
3. Removed the bones and resuspended the pellet in 500 µl FBS.
4. Centrifuged again at 956 × g for 6 minutes at 4°C and removed the supernatant.
5. Resuspended the pellet in 500 µl FBS and counted cells (4.5 million cells per tube).
6. Added 500 µl freezing medium and froze cells immediately in a Corning® CoolCell™ FTS30 at -80°C overnight.
7. Transferred cells to liquid nitrogen the next day.

Bait preparation

Due to its large size (S6P, 419 kDa), the SARS-CoV-2 prefusion-stabilized spike protein is likely subject to steric hindrance that prevents tetramer formation when complexed with streptavidin (55 kDa), whose biotin-binding sites are separated by 20 Å in cis and 35 Å in trans (1). Therefore, S6P and fluorochrome-conjugated streptavidin were combined at a 1:1 molar ratio to favor monomeric complexes. In contrast, the smaller SARS-CoV-2 receptor-binding domain (RBD, 28.2 kDa) likely forms tetramers and was thus used at a 4:1 molar ratio with streptavidin. Two fluorochromes were used per protein bait to improve signal to noise ratio and to clearly identify antigen reactive B cells (Figs. S4 and S5).

For the following steps, a total cell number of 3 million cells per sample was used.

RBD and S6P baits were freshly prepared the day before the staining experiment.

S6P preparation

The S6P bait contained trimeric SARS-CoV-2 spike protein, which was biotinylated using the Avitag™ technology (ACROBiosystems, Cat. SPN-C82E9). The biotinylated protein was labeled at a 1:1 ratio with the respective fluorochrome-coupled streptavidin. For each sample, 5 pmol S6P was added to either 5 pmol of BV480 or BV650-coupled streptavidin in a total volume of 25 µl.

1. The fluorochrome-coupled streptavidin tube was centrifuged at 10,000 x g for 1 minutes at 4°C to spin-down any free dye particles.

2. 5 pmol BV480-coupled streptavidin were added to 5 pmol S6P protein directly in Brilliant staining buffer.
3. 5 pmol BV650-coupled streptavidin to 5 pmol S6P protein were added directly to the Brilliant staining buffer.
4. The baits were incubated overnight at 4°C, protected from light.

RBD preparation

The RBD bait contains monomeric SARS-CoV-2 Spike RBD, biotinylated using the Avitag^{TM} technology (ACROBiosystems, Cat. SPD-C82E9). The biotinylated protein was labeled at a 4:1 ratio with a fluorochrome-coupled streptavidin (RB613 or RB744). For each sample, 10 pmol RBD was added to either RB613 or RB744-coupled streptavidin in a total volume of 25 µl.

For the staining, a 10 pmol bait solution for each dye in 25 µl/sample was prepared.

1. The fluorochrome-coupled streptavidin tube was centrifuged at 10,000 x g for 1 minutes at 4°C to spin-down any free dye particles.
2. In 4 increments with 20-minute incubations between each addition, 2.5 pmol RB613-coupled streptavidin was added to 10 pmol RBD protein directly in Brilliant staining buffer (2).
3. In 4 increments with 20-minute incubations between each addition, 2.5 pmol RB744-coupled streptavidin to 10 pmol RBD protein was added directly in Brilliant staining buffer.
4. Baits were incubated overnight at 4°C, protected from light.

Staining of single cell suspensions:

1. Thawed cells briefly in a 37°C water bath and transferred them immediately to a falcon containing 40 ml RPMI medium prewarmed to 37°C, allowing them to thaw completely.
2. Pelleted cells by centrifugation at 403 × g for 8 minutes at 4°C, aspirated the supernatant, and resuspended cells in 1 ml DPBS to remove any remaining proteins (e.g., FBS or bovine serum albumin) that could cause quenching of the free dye used for subsequent viability staining.
3. Counted cells and transferred 3×10^6 cells per staining into an Eppendorf tube.
4. Washed cells twice by adding 500 µl of DPBS, then centrifuged tubes at 956 × g for 3 minutes at 4°C.
5. Resuspended cells in freshly prepared 100 µl Live-Dead dye solution.
6. Incubated for 15 minutes at RT in the dark.
7. Washed cells by adding 500 µl of Staining Buffer to remove unbound viability dye, centrifuged tubes at 956 × g for 3 minutes at 4°C, and discarded the supernatant.
8. Resuspended cells in 100 µl of Fc block (diluted 1:100 - purified anti-mouse CD16/CD32, clone 93) in Staining Buffer and added 5 µl CellBlox™ blocking buffer per 100 µl cell sample containing 10^3 to 10^8 cells (to block non-specific binding of NovaFluor labels, PE, and APC tandems observed with macrophages and monocytes).
9. Incubated for 15 minutes at 4°C, protected from light.
10. Washed cells by adding 500 µl of Staining Buffer to remove unbound viability dye, centrifuged tubes at 956 × g for 3 minutes at 4°C, and discarded the supernatant.

11. Resuspended cells in 100 µl of final bait staining mix containing the final dilution of RBD and spike baits in Brilliant Staining Buffer.
12. Incubated for 1 hour at RT, protected from light.
13. Washed cells by adding 500 µl of Staining Buffer, centrifuged tubes at 956 × g for 3 minutes at 4°C, and discarded the supernatant.
14. Resuspended cells in 50 µl of antibody staining mix with the proper final dilutions of all antibodies diluted in Brilliant Staining Buffer.
15. Incubated for 30 minutes at 4°C, protected from light.
16. Washed cells twice by adding 500 µl of Staining Buffer per wash, centrifuged tubes at 956 × g for 3 minutes at 4°C, and discarded the supernatant.
17. Resuspended cells in 100 µl Staining Buffer and kept them in the dark at 4°C until analysis on the BD FACSymphony™ A5 SE.

Optimized T cell staining of single cell suspensions:

CD62L expression on T cells is highly sensitive to ex vivo handling and prone to enzymatic shedding during sample processing. In particular, temperature shifts and extended incubation at room temperature can lead to ADAM17-mediated cleavage of CD62L from the cell surface, reducing the accurate detection of naive and central memory T cells (3-6). To address this, we implemented a modified staining protocol using freshly prepared samples and minimized room-temperature exposure during processing, which resulted in robust and reproducible CD62L staining.

1. Dissected spleen from mice and transferred them into falcons with PBS.
2. Pressed tissue pieces through a 40 µm cell strainer into the same falcon filled with PBS using a syringe plunger.
3. Pelleted cells by centrifugation at 403 × g for 10 minutes at 4°C, aspirated the supernatant, and resuspended cells in 1 ml DPBS to remove any remaining proteins that could cause quenching of the free dye used for subsequent viability staining.
4. Counted cells and transferred 3×10^6 cells per staining into an Eppendorf tube.
5. Washed cells twice by adding 500 µl of DPBS, then centrifuged tubes at 956 × g for 3 minutes at 4°C.
6. Resuspended cells in freshly prepared 100 µl Live-Dead dye solution.
7. Incubated for 30 minutes at 4°C in the dark.
8. Washed cells by adding 500 µl of Staining Buffer to remove unbound viability dye, centrifuged tubes at 956 × g for 3 minutes at 4°C, and discarded the supernatant.
9. Resuspended cells in 100 µl of Fc block (diluted 1:100 - purified anti-mouse CD16/CD32, clone 93) in Staining Buffer and added 5 µl CellBlox™ blocking buffer per 100 µl cell sample containing 10^3 to 10^8 cells (to block non-specific binding of NovaFluor labels, PE, and APC tandems observed with macrophages and monocytes).
10. Incubated for 15 minutes at 4°C, protected from light.
11. Washed cells by adding 500 µl of Staining Buffer to remove unbound viability dye, centrifuged tubes at 956 × g for 3 minutes at 4°C, and discarded the supernatant.
12. Resuspended cells in 50 µl of antibody staining mix with the proper final dilutions of all antibodies diluted in Brilliant Staining Buffer.
13. Incubated for 30 minutes at 4°C, protected from light.

14. Washed cells twice by adding 500 µl of Staining Buffer per wash, centrifuged tubes at 956 × g for 3 minutes at 4°C, and discarded the supernatant.
15. Resuspended cells in 100 µl Staining Buffer and kept them in the dark at 4°C until analysis on the BD FACSymphony™ A5 SE.

Staining workflow for mouse lymphoid panel

	Marker	Fluorochrome	Dilution	Diluent	Incubation (min/Temp)
1	Fixable Viability Stain 440UV		1:500	DPBS	15'/RT
2	**Wash (Staining buffer)**				
3	Fc Block	-	1:100	Staining Buffer	15'/4°C
	CellBlox™ blocking buffer	-	1:20		
4	**Wash (Staining buffer)**				
5	S6P	BV480	5 pmol	Brilliant Stain Buffer	60'/RT
	S6P	BV650	5 pmol		
	RBD	RB613	10 pmol		
	RBD	RB744	10 pmol		
6	**Wash (Staining buffer)**				
7	B220	BUV395	1:100	Brilliant Stain Buffer	30'/4°C
	CD38	BUV563	1:200		
	CD80	BUV615	1:100		
	Ep-CAM	BUV661	1:100		
	CD21	BUV737	1:1600		
	CD23	BUV805	1:200		
	TACI	BV421	1:100		
	CD44	V450	1:400		
	IgG1	BV510	1:100		
	IgG2a/b	BV510	1:100		
	IgG3	BV510	1:100		
	PD-1	BV605	1:100		
	CXCR3	BV711	1:100		
	CD138	BV750	1:400		
	IgA	FITC	1:200		
	CD86	RB545	1:100		
	CD62L	RB705	1:1000		
	CD19	RB780	1:800		
	CD93	RY586	1:100		
	CXCR5	PE/Dazzle 594	1:200		
	IgM	NovaFluor™ Yellow 660	1:100		
	CD8a	PE-Cy5.5	1:400		
	TIGIT	PE-Cy7	1:100		

	Marker	Fluorochrome	Dilution	Diluent	Incubation (min/Temp)
	GL7	AF647	1:100		
	NK-1.1	APC	1:100		
	TER-119	APC	1:100		
	F4/80	APC	1:200		
	Ly-6G/Ly-6C (Gr-1)	APC	1:100		
	CD11c	APC	1:100		
	Ly6A	R718	1:400		
	IgD	APC-H7	1:200		
	CD4	APC-H7	1:400		

**8**	**Wash (Staining buffer)**

**9**	**Resuspend cells in 100 µl of Staining buffer and keep in dark at 4°C until acquisition (max 3 hours)**

Single-Color Reference Control

Correct spectral unmixing was confirmed using single-stained cells. For spectral unmixing, single-color reference controls for each marker were prepared using either cells or beads, including splenocytes, bone marrow-derived lymphocytes, and UltraComp eBeads™ Compensation Beads. Due to the limited number of lymphocytes obtained per sample, particularly when analyzing rare B and T cell subsets across multiple tissues, it was not feasible to allocate sufficient cells to prepare single-color controls for every fluorochrome. We therefore aimed to generate the majority of single-color reference controls on beads. To validate this approach, we conducted a comparative experiment assessing the quality of spectral unmixing between controls prepared on beads and those on cells. If beads provided comparable unmixing accuracy, they were selected as reference controls (Supplementary Table S4).

The quality of spectral unmixing was evaluated using NxN plots, which display pairwise comparisons of all fluorochromes across detectors. To this end, all single-color reference controls were pooled and acquired together. As each sample contained only one labeled marker, we expected to observe exclusively single-positive populations. Successful spectral unmixing was defined by the presence of clearly separated single-positive signals and minimal spillover into other channels (Figure S2). For antigen baits, we applied a surrogate staining strategy, using antibodies targeting abundantly expressed surface markers that were conjugated to the same fluorochrome and obtained from the same manufacturer, which yielded satisfactory unmixing results. Additionally, the separation of cell populations was further improved by applying autofluorescence extraction.

Cell-based staining protocol for single-color reference controls

1. Up to $2x10^6$ cells per single-color reference control were used. Cells were pelleted by centrifugation at 956 x g for 3 minutes at 4°C in Eppendorf tubes.
2. Resuspend cells in 100 µl Staining Buffer and add 5 µl CellBlox™ blocking buffer/ 100 µl cell single color reference control containing 10^3 to 10^8 cells (to block non-specific binding of NovaFluor labels, PE and APC tandems observed with macrophages and monocytes) where needed and incubate for 15 minutes at 4°C.
3. Washed cells by adding 500 µl of Staining buffer, centrifuge tubes at 956 x g for 3 minutes at 4°C and discard supernatant.
4. Resuspend cells in 100 µl of predetermined amount of antibody diluted in Brilliant staining buffer.
5. Incubated for 30 minutes at 4°C, protected from light.
6. Washed cells by adding 500 µl of Staining buffer, centrifuge tubes at 956 x g for 3 minutes at 4°C and discard supernatant.
7. Resuspend cells in 100 µl Staining buffer and keep in dark at 4°C until acquisition.
8. Collected 500 events within the negative and positive gates of interest.

Bead-based single color reference controls

The UltraComp eBeads™ Compensation Beads (Invitrogen, Cat. #01-2222-42) contained two bead populations. The positive bead population captured the fluorochrome-conjugated antibody used for cell staining, whereas the negative bead population did not bind the antibody.

1. One drop of UltraComp eBeads™ Compensation Beads was placed into an Eppendorf tube.
2. Respective antibody diluted in Brilliant staining buffer (100 µl) was added.
3. Beads were incubated for 30 minutes at 4°C, protected from light.
4. Beads were washed by adding 500 µl staining buffer, centrifuged at 956 × g for 3 minutes at 4°C, and the supernatant was discarded.
5. Beads were resuspended in 100 µl staining buffer and stored in the dark at 4°C until acquisition.
6. 5000 events within the gate of interest were recorded.

References

1. Fairhead M, Krndija D, Lowe ED, Howarth M. Plug-and-play pairing via defined divalent streptavidins. J Mol Biol. 2014;426(1):199-214.
2. Pape KA, Dileepan T, Kabage AJ, Kozysa D, Batres R, Evert C, et al. High-affinity memory B cells induced by SARS-CoV-2 infection produce more plasmablasts and atypical memory B cells than those primed by mRNA vaccines. Cell Rep. 2021;37(2):109823.
3. Kapitza L, Ho N, Kerzel T, Frank AM, Thalheimer FB, Jamali A, et al. CD62L as target receptor for specific gene delivery into less differentiated human T lymphocytes. Front Immunol. 2023;14:1183698.
4. Savage ND, Harris SH, Rossi AG, De Silva B, Howie SE, Layton GT, et al. Inhibition of TCR-mediated shedding of L-selectin (CD62L) on human and mouse CD4+ T cells by metalloproteinase inhibition: analysis of the regulation of Th1/Th2 function. Eur J Immunol. 2002;32(10):2905-14.
5. Sopper S, Mustjoki S, White D, Hughes T, Valent P, Burchert A, et al. Reduced CD62L Expression on T Cells and Increased Soluble CD62L Levels Predict Molecular Response to Tyrosine Kinase Inhibitor Therapy in Early Chronic-Phase Chronic Myelogenous Leukemia. J Clin Oncol. 2017;35(2):175-84.
6. Yang S, Liu F, Wang QJ, Rosenberg SA, Morgan RA. The shedding of CD62L (L-selectin) regulates the acquisition of lytic activity in human tumor reactive T lymphocytes. PLoS One. 2011;6(7):e22560.

Supplementary Information | Figures

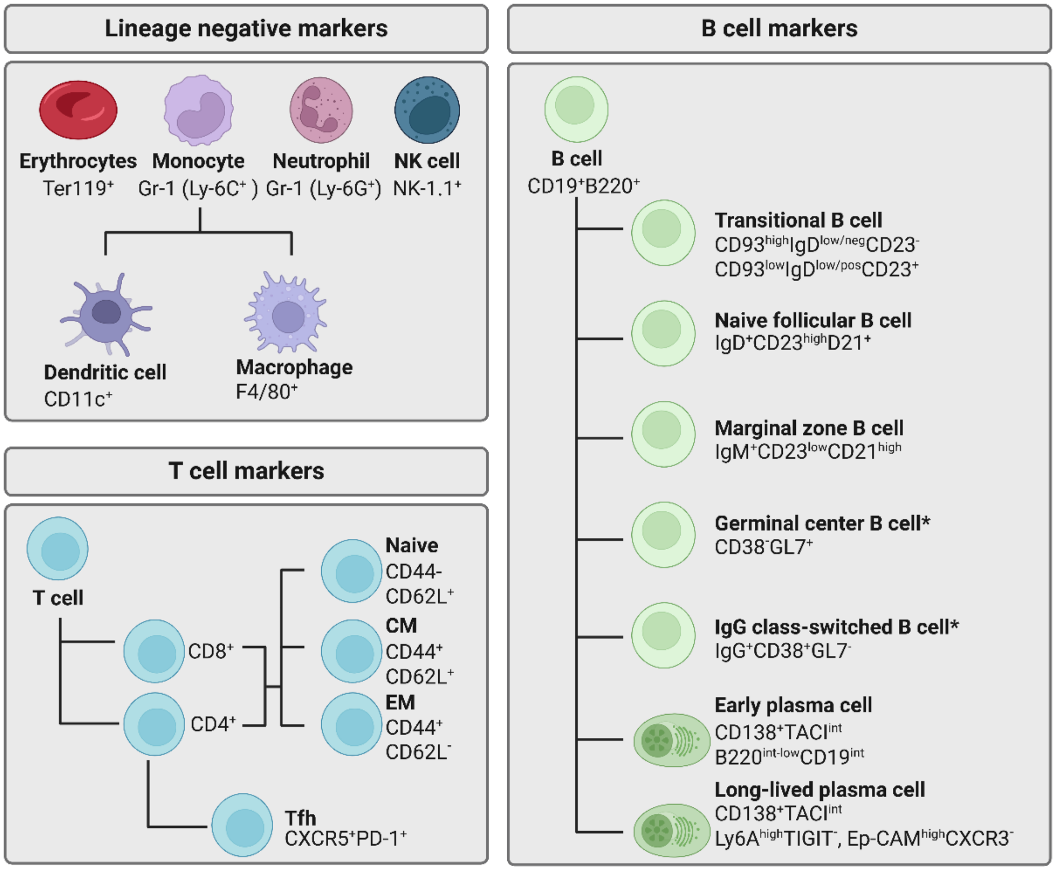

Supplementary Figure S1: Overview of different lymphoid and myeloid subsets that can be discriminated with the panel. Phenotypic markers used to discriminate lymphoid cell populations including antigen-reactive B cells (subsets marked with an asterisk were analyzed) (1-7). CM: Central memory, EM: Effector memory, Tfh: T follicular helper cell. Created in https://BioRender.com

A

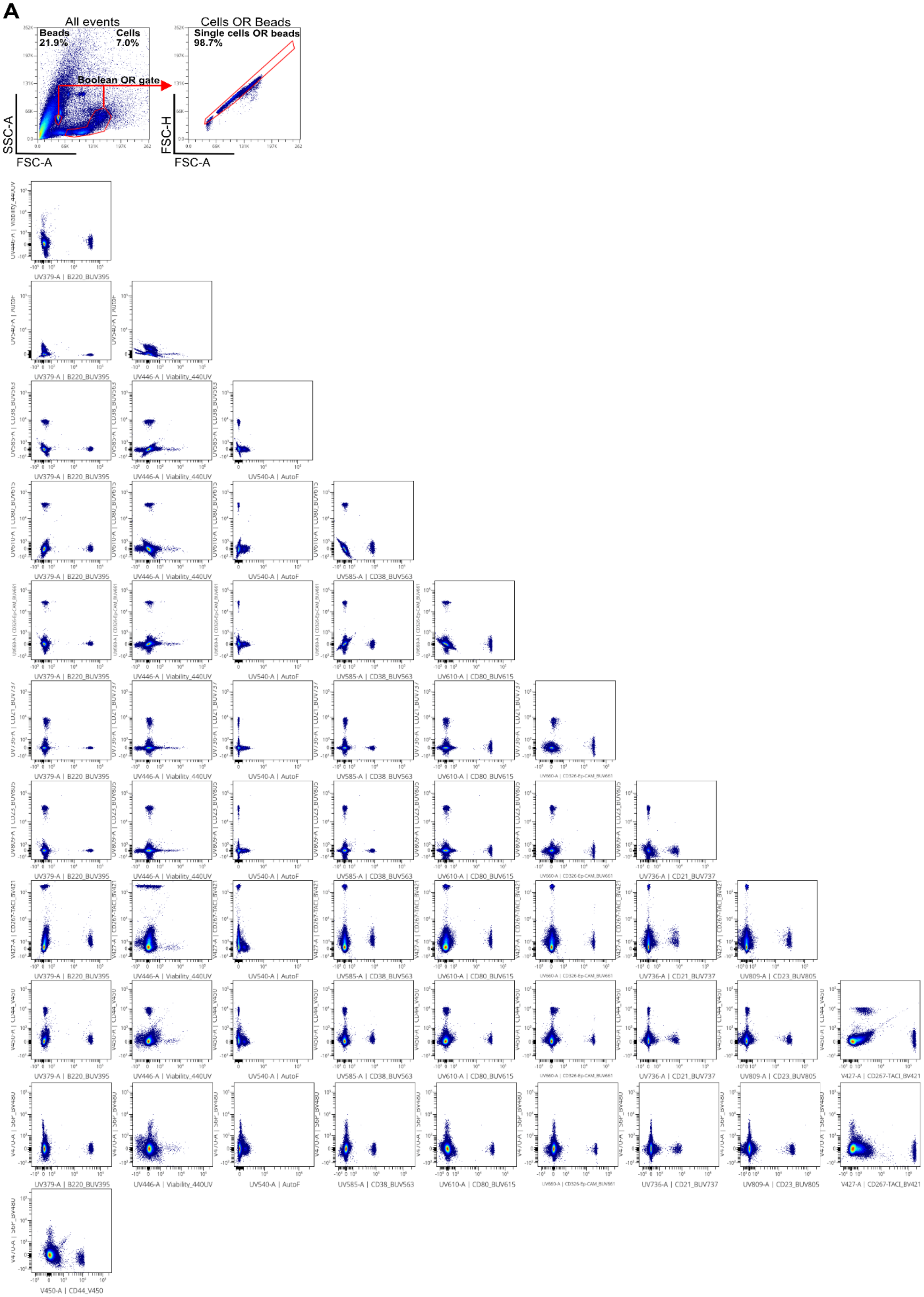
All events
Beads 21.9%
Cells 7.0%
Boolean OR gate
SSC-A
FSC-A
Cells OR Beads
Single cells OR beads 98.7%
FSC-H
FSC-A
UV379-A | B220_BUV395
UV446-A | Viability_440UV
UV540-A | AutoF
UV585-A | CD38_BUV563
UV610-A | CD80_BUV615
UV736-A | CD21_BUV737
UV809-A | CD23_BUV805
V427-A | CD267-TACI_BV421
V450-A | CD44_V450

B

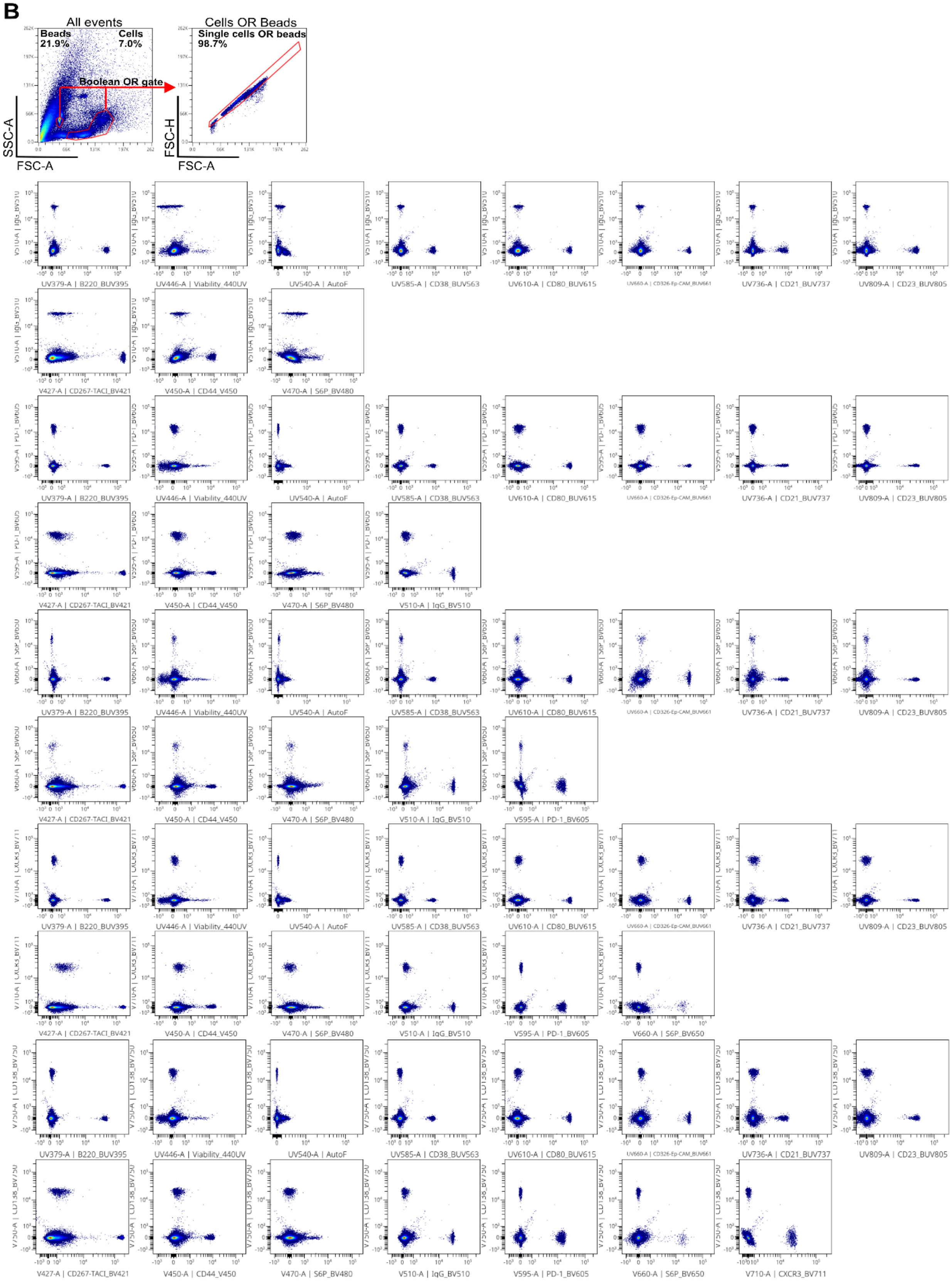
All events
Beads 21.9%
Cells 7.0%
Boolean OR gate
SSC-A
FSC-A
Cells OR Beads
Single cells OR beads 98.7%
FSC-H
FSC-A
UV379-A | B220_BUV395
UV446-A | Viability_440UV
UV540-A | AutoF
UV585-A | CD38_BUV563
UV610-A | CD80_BUV615
UV660-A | CD326-Ep-CAM_BUV661
UV736-A | CD21_BUV737
UV809-A | CD23_BUV805
V427-A | CD267-TACI_BV421
V450-A | CD44_V450
V470-A | S6P_BV480
V510-A | IgG_BV510
V595-A | PD-1_BV605
V660-A | S6P_BV650
V710-A | CXCR3_BV711

C

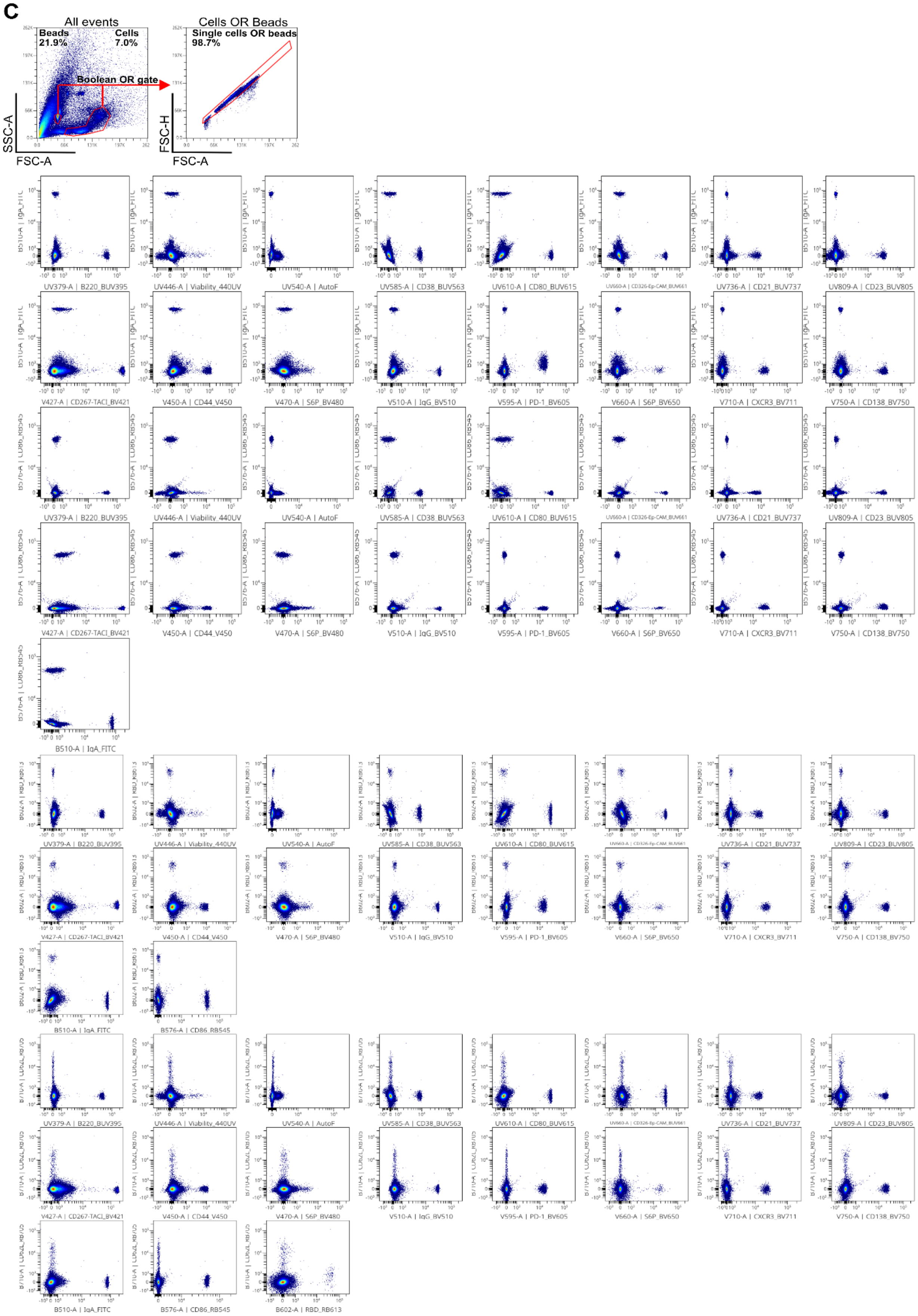
All events
Beads 21.9%
Cells 7.0%
Boolean OR gate
SSC-A
FSC-A
Cells OR Beads
Single cells OR beads 98.7%
FSC-H
FSC-A
UV379-A | B220_BUV395
UV446-A | Viability_440UV
UV540-A | AutoF
UV585-A | CD38_BUV563
UV610-A | CD80_BUV615
UV660-A | CD326-Ep-CAM_BUV661
UV736-A | CD21_BUV737
UV809-A | CD23_BUV805
V427-A | CD267-TACI_BV421
V450-A | CD44_V450
V470-A | S6P_BV480
V510-A | IgG_BV510
V595-A | PD-1_BV605
V660-A | S6P_BV650
V710-A | CXCR3_BV711
V750-A | CD138_BV750
B510-A | IgA_FITC
B576-A | CD86_RB545
B602-A | RBD_RB613

D

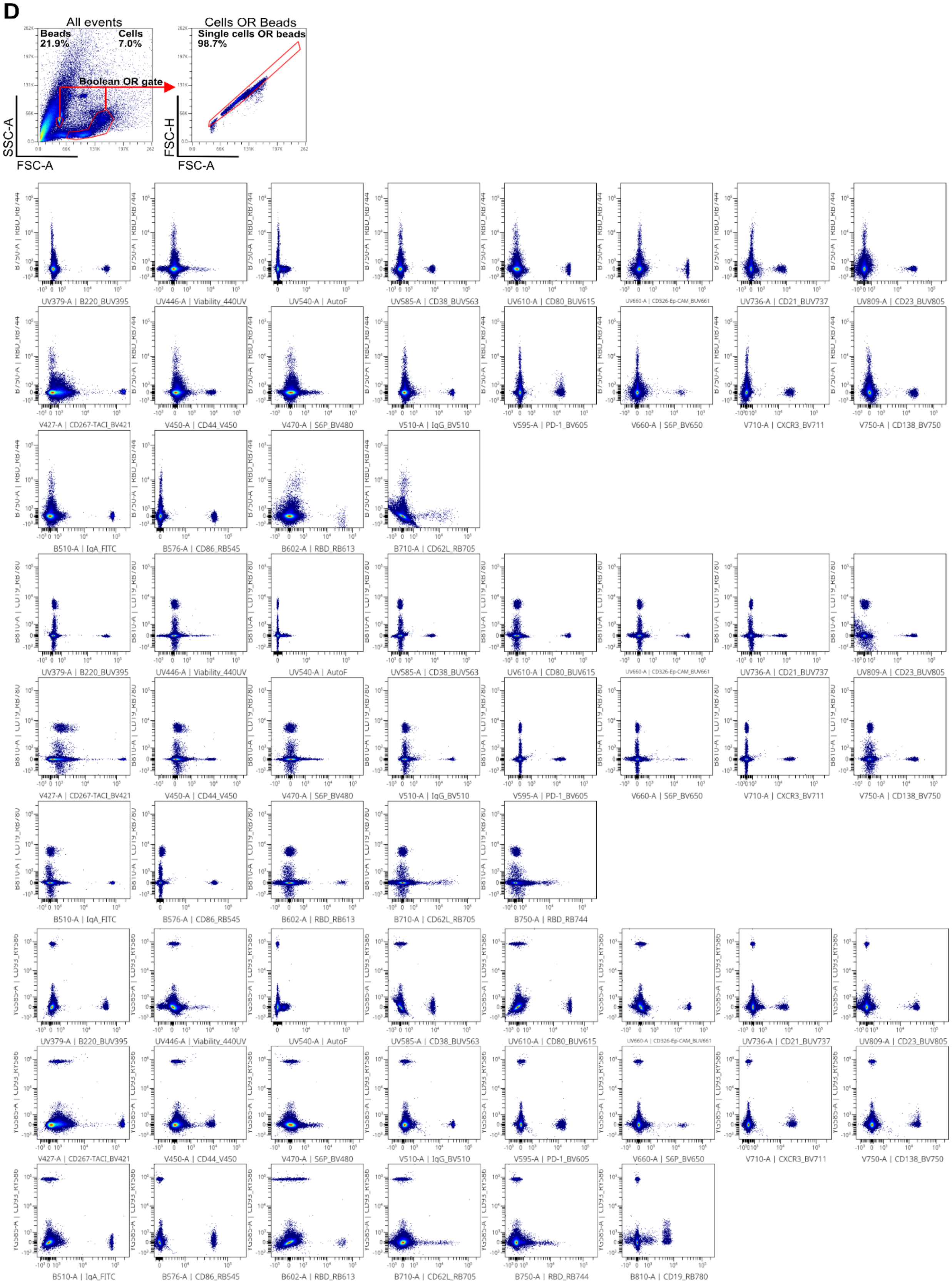
All events
Beads 21.9%
Cells 7.0%
Boolean OR gate
SSC-A
FSC-A
Cells OR Beads
Single cells OR beads 98.7%
FSC-H
FSC-A
UV379-A | B220_BUV395
UV446-A | Viability_440UV
UV540-A | AutoF
UV585-A | CD38_BUV563
UV610-A | CD80_BUV615
UV660-A | CD326-Ep-CAM_BUV661
UV736-A | CD21_BUV737
UV809-A | CD23_BUV805
V427-A | CD267-TACI_BV421
V450-A | CD44_V450
V470-A | S6P_BV480
V510-A | IgG_BV510
V595-A | PD-1_BV605
V660-A | S6P_BV650
V710-A | CXCR3_BV711
V750-A | CD138_BV750
B510-A | IgA_FITC
B576-A | CD86_RB545
B602-A | RBD_RB613
B710-A | CD62L_RB705
B750-A | RBD_RB744
B810-A | CD19_RB780

E

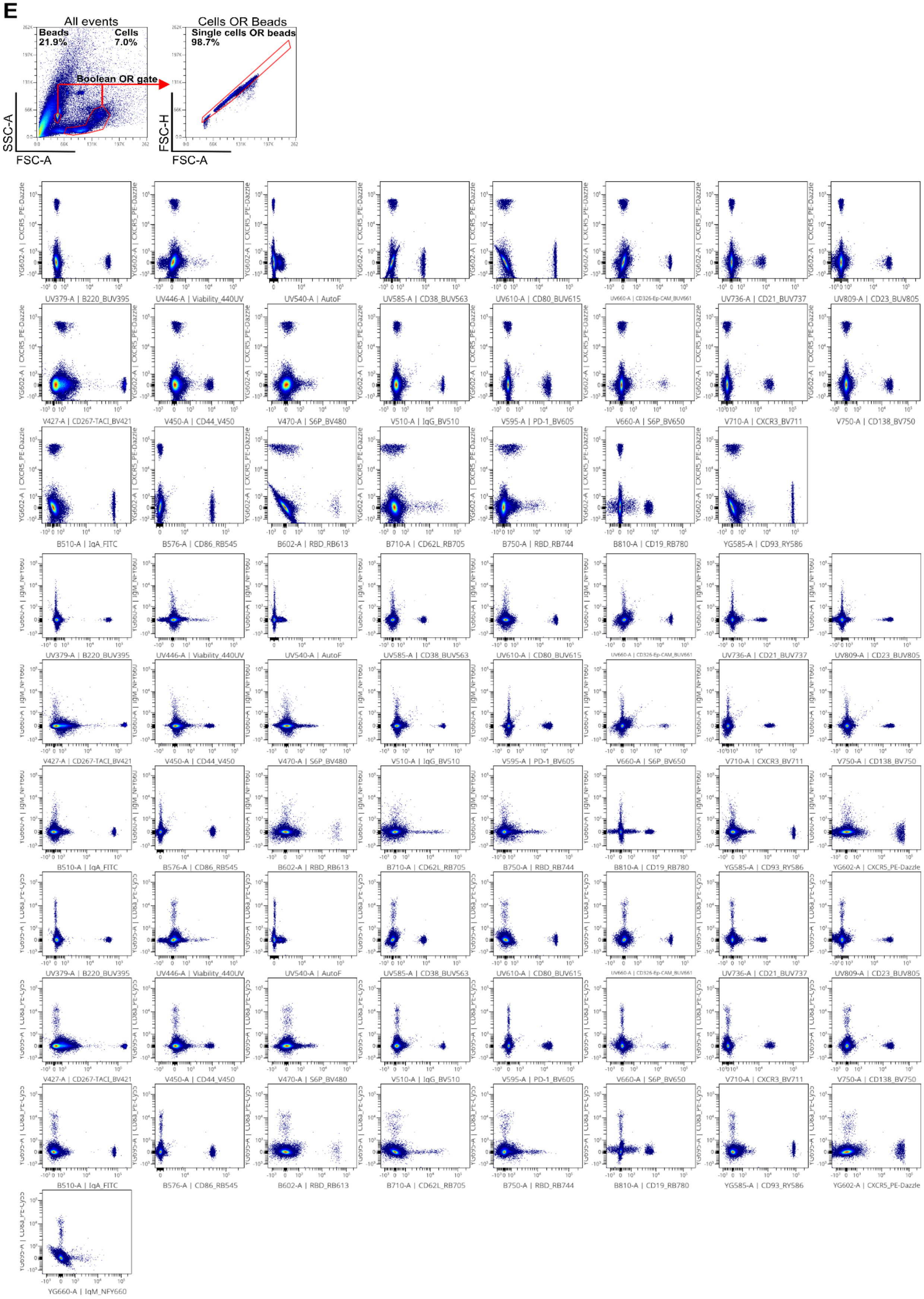
All events
Beads 21.9%
Cells 7.0%
Boolean OR gate
SSC-A
FSC-A
Cells OR Beads
Single cells OR beads 98.7%
FSC-H
FSC-A
YG602-A | CXCR5_PE-Dazzle
UV379-A | B220_BUV395
UV446-A | Viability_440UV
UV540-A | AutoF
UV585-A | CD38_BUV563
UV610-A | CD80_BUV615
UV660-A | CD326-Ep-CAM_BUV661
UV736-A | CD21_BUV737
UV809-A | CD23_BUV805
V427-A | CD267-TACI_BV421
V450-A | CD44_V450
V470-A | S6P_BV480
V510-A | IgG_BV510
V595-A | PD-1_BV605
V660-A | S6P_BV650
V710-A | CXCR3_BV711
V750-A | CD138_BV750
B510-A | IgA_FITC
B576-A | CD86_RB545
B602-A | RBD_RB613
B710-A | CD62L_RB705
B750-A | RBD_RB744
B810-A | CD19_RB780
YG585-A | CD93_RY586
UV379-A | B220_BUV395
UV446-A | Viability_440UV
UV540-A | AutoF
UV585-A | CD38_BUV563
UV610-A | CD80_BUV615
UV660-A | CD326-Ep-CAM_BUV661
UV736-A | CD21_BUV737
UV809-A | CD23_BUV805
V427-A | CD267-TACI_BV421
V450-A | CD44_V450
V470-A | S6P_BV480
V510-A | IgG_BV510
V595-A | PD-1_BV605
V660-A | S6P_BV650
V710-A | CXCR3_BV711
V750-A | CD138_BV750
B510-A | IgA_FITC
B576-A | CD86_RB545
B602-A | RBD_RB613
B710-A | CD62L_RB705
B750-A | RBD_RB744
B810-A | CD19_RB780
YG585-A | CD93_RY586
YG602-A | CXCR5_PE-Dazzle
UV379-A | B220_BUV395
UV446-A | Viability_440UV
UV540-A | AutoF
UV585-A | CD38_BUV563
UV610-A | CD80_BUV615
UV660-A | CD326-Ep-CAM_BUV661
UV736-A | CD21_BUV737
UV809-A | CD23_BUV805
V427-A | CD267-TACI_BV421
V450-A | CD44_V450
V470-A | S6P_BV480
V510-A | IgG_BV510
V595-A | PD-1_BV605
V660-A | S6P_BV650
V710-A | CXCR3_BV711
V750-A | CD138_BV750
B510-A | IgA_FITC
B576-A | CD86_RB545
B602-A | RBD_RB613
B710-A | CD62L_RB705
B750-A | RBD_RB744
B810-A | CD19_RB780
YG585-A | CD93_RY586
YG602-A | CXCR5_PE-Dazzle
YG660-A | IgM_NFY660

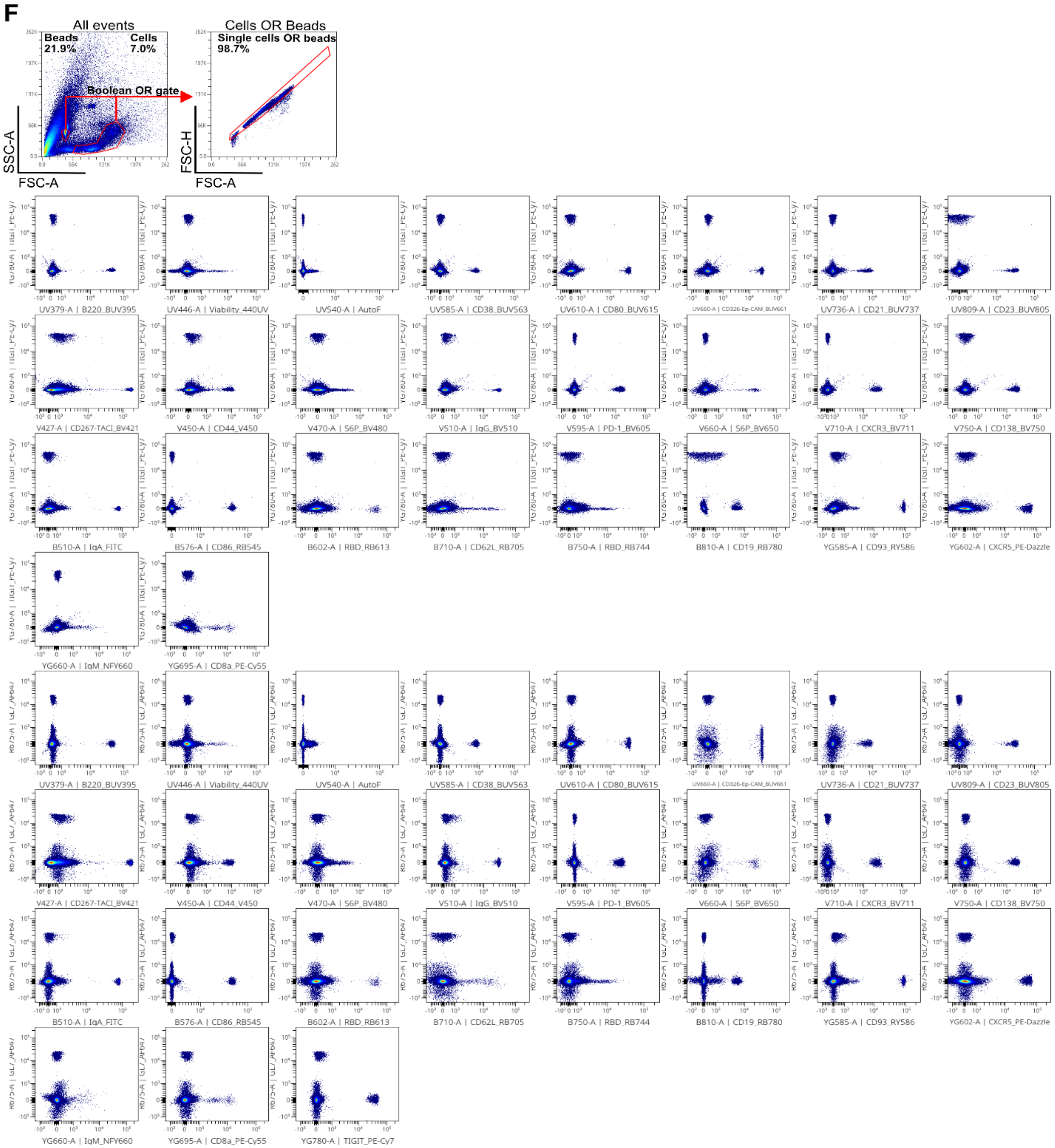

Supplementary Figure S2: (A–F) Quality control of spectral unmixing using NxN plots of single-color reference controls. Beads and cells were first gated individually and then combined using a Boolean OR gate to include all relevant events. A subsequent gate was applied to exclude doublets and aggregates. NxN plots display pairwise comparisons of fluorochromes across all detectors. Successful spectral unmixing is indicated by the presence of distinct single-positive populations with minimal signal spillover into other channels. Data shown are representative of three independent single-color reference control (*n* = 3).

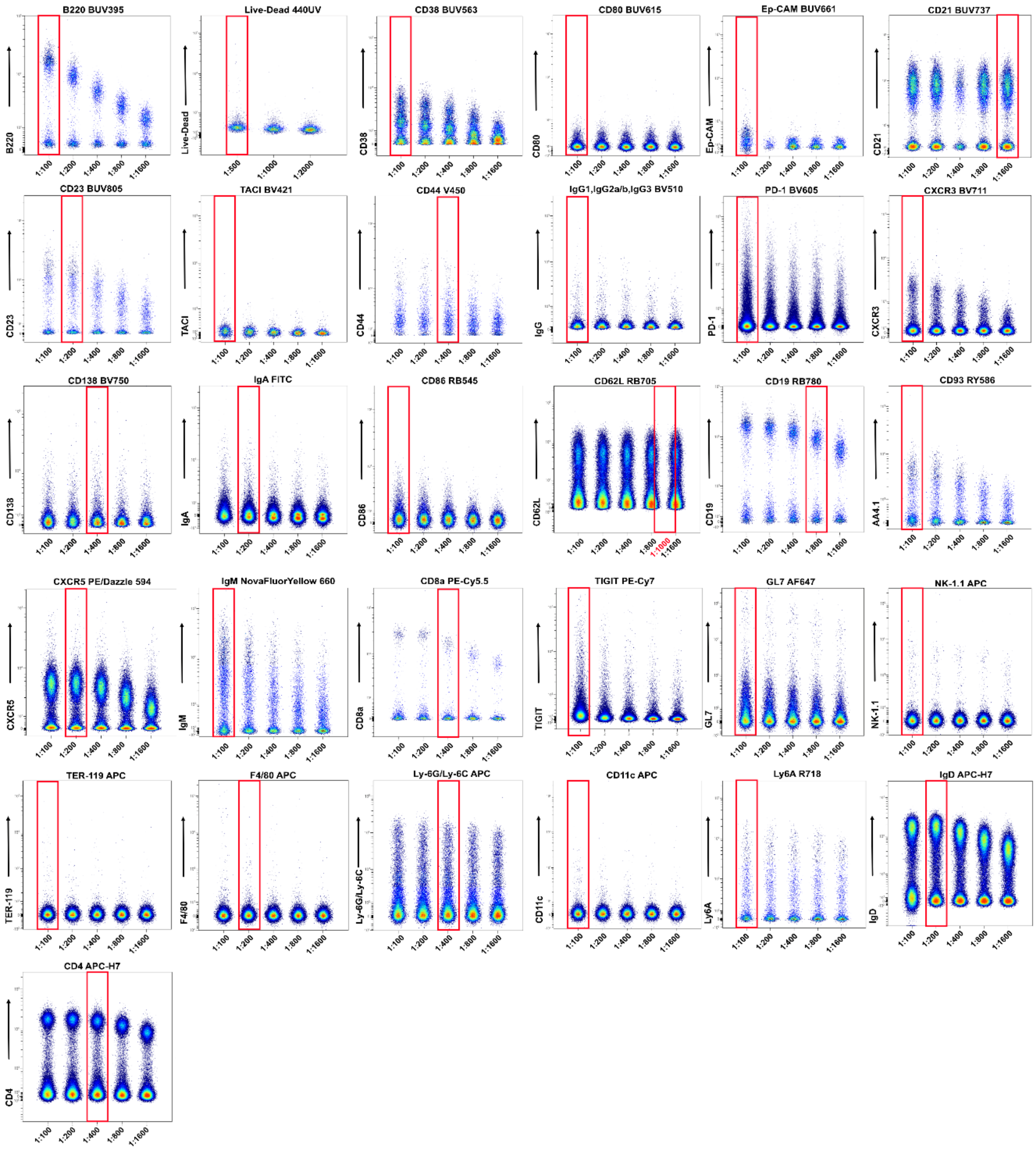

Supplementary Figure S3: Antibody titrations for panel optimization. Each antibody was tested in five 2-fold serial dilutions (indicated on the x-axis). Optimal antibody concentrations were defined as the lowest dilution providing maximal signal separation with minimal background staining. Red frames highlight dilutions used in the final panel. Data shown are representative of three mice (*n* = 3).

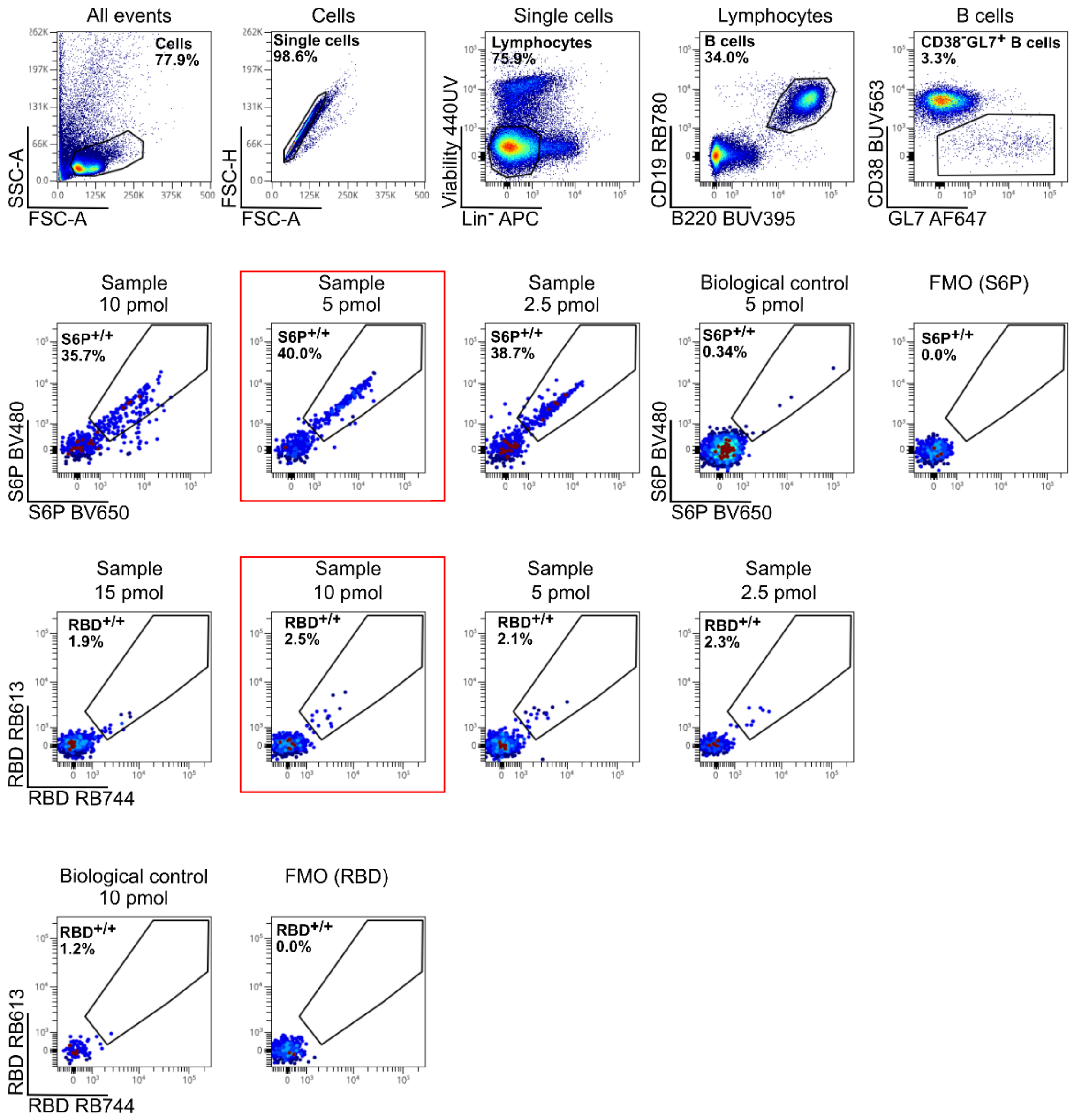

Supplementary Figure S4: SARS-CoV-2 spike protein (S6P) and receptor binding domain (RBD) bait titration. Optimal bait concentrations were defined as the lowest amount yielding the best signal-to-noise separation. Negative controls included cells from mice immunized with adjuvant only (biological control) as well as FMO controls. Red frames highlight the bait concentrations selected for use in the final panel. Data shown are representative of six mice (*n* = 6).

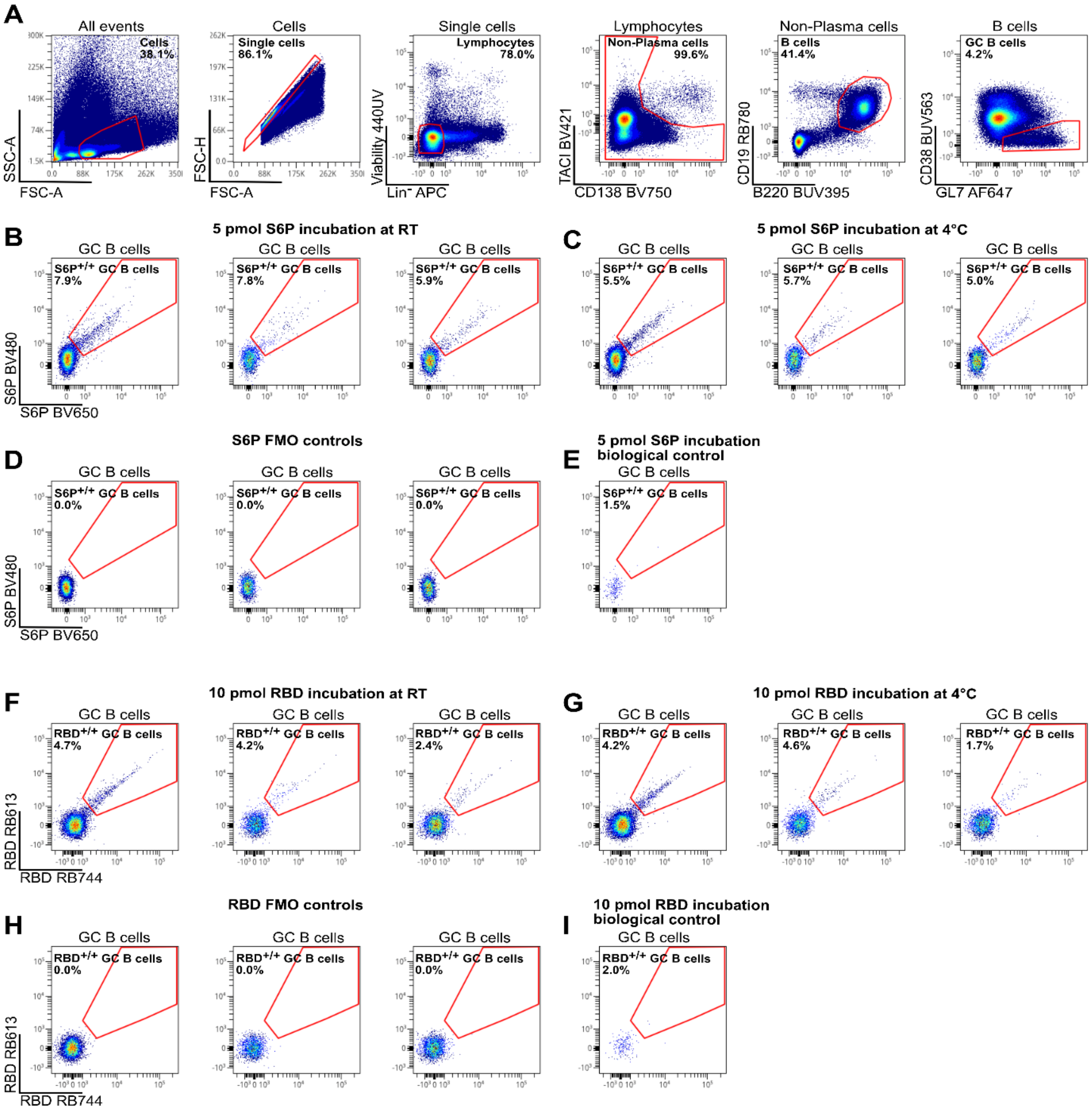

Supplementary Figure S5: SARS-CoV-2 spike protein (S6P) and receptor binding domain (RBD) bait staining was tested under different incubation conditions. To determine the optimal staining conditions for identifying low-frequency antigen-reactive B cells, bait staining was evaluated at room temperature and at 4 °C. **(A)** Gating strategy for viable lymphocytes following the exclusion of cellular debris, doublets, dead cells, and lineage-negative (Lin^{-}) cells (TER^{-}119^{+}, Ly-6G/Ly-6C^{+} [Gr-1^{+}], F4/80^{+}, CD11c^{+}, NK-1.1^{+}). Plasma cells (PCs) and plasmablasts (PBs; TACI^{int}CD138^{+}) were excluded from B cells (CD19^{+}B220^{+}). Germinal center (GC) B cells (CD38^{-}GL7^{+}) were identified. **(B, F)** Preincubation with the respective bait for 60 minutes at room temperature. **(C, G)** Preincubation with the respective bait for 60 minutes at 4°C. **(D)** S6P BV40 and S6P BV650 FMO staining controls. **(E, I)** Preincubation of a sample from a non-immunized mouse with the S6P (E) or RBD (I) bait for 60 minutes at room temperature. **(H)** RBD RB613 and RBD RB744 FMO staining controls. For the final panel, all antigen bait stainings were performed for 60 minutes at room temperature. Data shown are representative of three mice (*n* = 3).

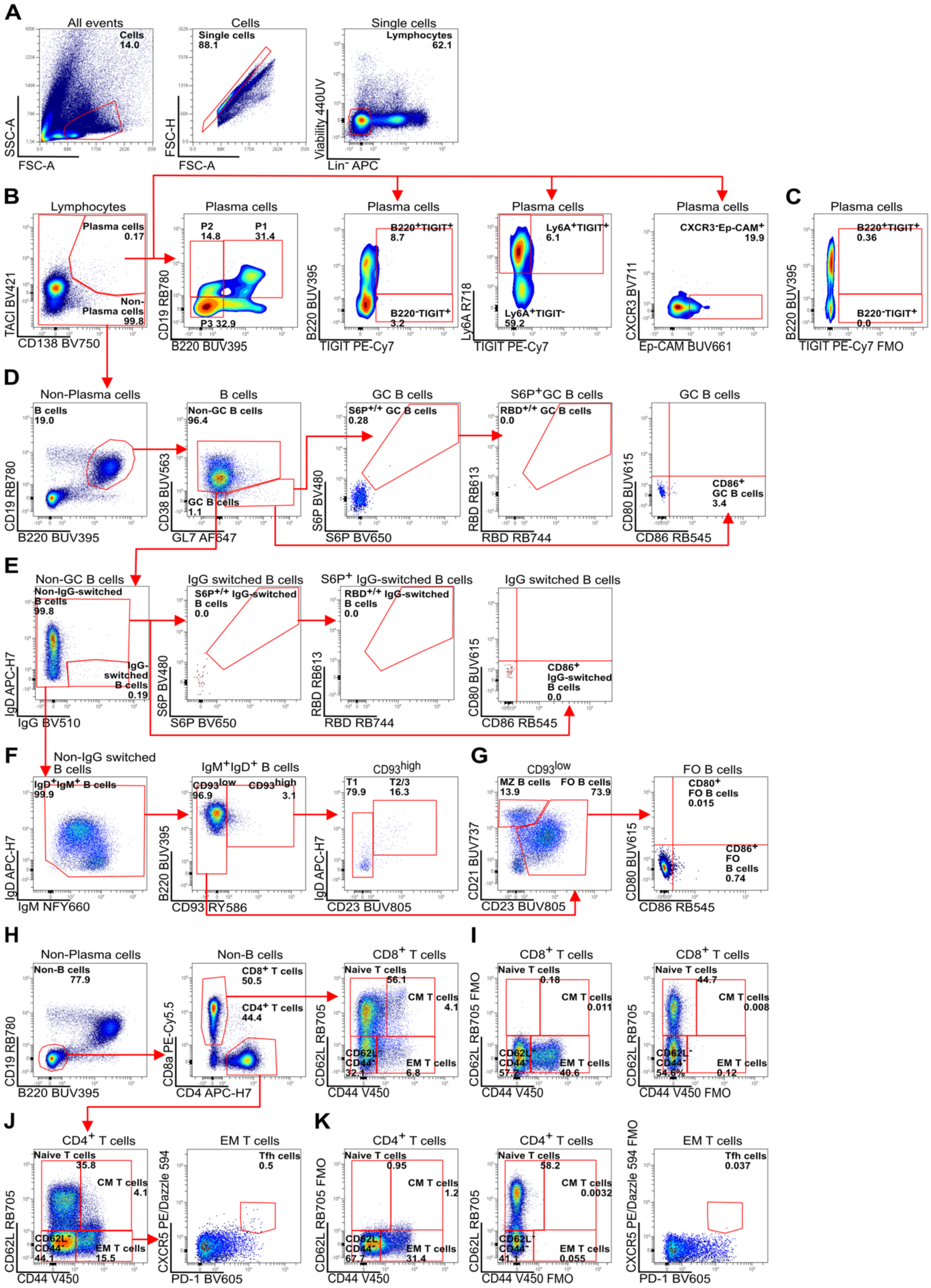
A
All events
Cells 14.0
SSC-A
FSC-A
Cells
Single cells 88.1
FSC-H
FSC-A
Single cells
Lymphocytes 62.1
Viability 440UV
Lin⁻ APC
B
Lymphocytes
Plasma cells 0.17
Non-Plasma cells 99.8
TACI BV421
CD138 BV750
Plasma cells
P2 14.8
P1 31.4
P3 32.9
CD19 RB780
B220 BUV395
Plasma cells
B220⁺TIGIT⁺ 8.7
B220⁻TIGIT⁺ 3.2
B220 BUV395
TIGIT PE-Cy7
Plasma cells
Ly6A⁺TIGIT⁺ 6.1
Ly6A⁺TIGIT⁻ 59.2
Ly6A R718
TIGIT PE-Cy7
Plasma cells
CXCR3⁻Ep-CAM⁺ 19.9
CXCR3 BV711
Ep-CAM BUV661
C
Plasma cells
B220⁺TIGIT⁺ 0.36
B220⁻TIGIT⁺ 0.0
B220 BUV395
TIGIT PE-Cy7 FMO
D
Non-Plasma cells
B cells 19.0
CD19 RB780
B220 BUV395
B cells
Non-GC B cells 96.4
GC B cells 1.1
CD38 BUV563
GL7 AF647
GC B cells
S6P⁺/⁺ GC B cells 0.28
S6P BV480
S6P BV650
S6P⁺GC B cells
RBD⁺/⁺ GC B cells 0.0
RBD RB613
RBD RB744
GC B cells
CD86⁺ GC B cells 3.4
CD80 BUV615
CD86 RB545
E
Non-GC B cells
Non-IgG-switched B cells 99.8
IgG-switched B cells 0.19
IgD APC-H7
IgG BV510
IgG switched B cells
S6P⁺/⁺ IgG-switched B cells 0.0
S6P BV480
S6P BV650
S6P⁺ IgG-switched B cells
RBD⁺/⁺ IgG-switched B cells 0.0
RBD RB613
RBD RB744
IgG switched B cells
CD86⁺ IgG-switched B cells 0.0
CD80 BUV615
CD86 RB545
F
Non-IgG switched B cells
IgD⁺IgM⁺ B cells 99.9
IgD APC-H7
IgM NFY660
IgM⁺IgD⁺ B cells
CD93low 96.9
CD93high 3.1
B220 BUV395
CD93 RY586
CD93high
T1 79.9
T2/3 16.3
IgD APC-H7
CD23 BUV805
G
CD93low
MZ B cells 13.9
FO B cells 73.9
CD21 BUV737
CD23 BUV805
FO B cells
CD80⁺ FO B cells 0.015
CD86⁺ FO B cells 0.74
CD80 BUV615
CD86 RB545
H
Non-Plasma cells
Non-B cells 77.9
CD19 RB780
B220 BUV395
Non-B cells
CD8⁺ T cells 50.5
CD4⁺ T cells 44.4
CD8a PE-Cy5.5
CD4 APC-H7
CD8⁺ T cells
Naive T cells 56.1
CM T cells 4.1
CD62L⁻CD44⁻ 32.1
EM T cells 6.8
CD62L RB705
CD44 V450
I
CD8⁺ T cells
Naive T cells 0.18
CM T cells 0.011
CD62L⁻CD44⁻ 57.2
EM T cells 40.6
CD62L RB705 FMO
CD44 V450
CD8⁺ T cells
Naive T cells 44.7
CM T cells 0.008
CD62L⁻CD44⁻ 54.6%
EM T cells 0.12
CD62L RB705
CD44 V450 FMO
J
CD4⁺ T cells
Naive T cells 35.8
CM T cells 4.1
CD62L⁻CD44⁻ 44.1
EM T cells 15.5
CD62L RB705
CD44 V450
EM T cells
Tfh cells 0.5
CXCR5 PE/Dazzle 594
PD-1 BV605
K
CD4⁺ T cells
Naive T cells 0.95
CM T cells 1.2
CD62L⁻CD44⁻ 67.7
EM T cells 31.4
CD62L RB705 FMO
CD44 V450
CD4⁺ T cells
Naive T cells 58.2
CM T cells 0.0032
CD62L⁻CD44⁻ 41.1
EM T cells 0.055
CD62L RB705
CD44 V450 FMO
EM T cells
Tfh cells 0.037
CXCR5 PE/Dazzle 594 FMO
PD-1 BV605

Supplementary Figure S6: Gating strategy for murine spleen samples from a *non*-immunized mouse. (A) Gating strategy for viable lymphocytes following the exclusion of cellular debris, doublets, dead cells, and lineage-negative (Lin^-) cells (TER^-119^+, Ly-6G/Ly-$6C^+$ [Gr-1^+], F4/80^+, $CD11c^+$, NK-1.1^+). **(B)** Plasma cells (PCs) and plasmablasts (PBs; $TACI^{int}CD138^+$) were further subdivided into three subsets: P1 – PC precursors ($B220^{int}CD19^{int}$), P2 – early PCs ($B220^{lo}CD19^{int}$), and P3 – mature PCs ($B220^{low}CD19^{low}$). PCs expressing Ep-$CAM^{high}CXCR3^-$ or $Ly6A^{high}TIGIT^-$ phenotypes were identified. **(C)** TIGIT PE-Cy7 FMO staining control. **(D)** Germinal center (GC) B cells ($CD38^-GL7^+$) were investigated for antigen reactivity ($S6P^{+/+}$, $RBD^{+/+}$) as well as their activation status ($CD86^+$). **(E)** IgG^+ class-switched B cells ($CD38^+GL7^-IgG^+$) showed no antigen-reactivity ($S6P^{+/+}$, $RBD^{+/+}$) and did not express the activation marker CD86. **(F)** T1-stage transitional B cells ($IgM^{high}CD23^{low}$) and T2-stage transitional B cells ($IgD^{high}CD23^{high}$) were identified. **(G)** Naive follicular ($CD23^{high}CD21^+$) and marginal zone ($CD23^{low}CD21^{high}$) B cells were defined. Activated naive follicular B cells ($CD80^+$) were characterized. **(H)** $CD8^+$ T cells were divided into naive $CD8^+$ T cells ($CD44^-$ $CD62L^+$), central memory (CM) $CD8^+$ T cells ($CD44^+CD62L^+$), and effector memory (EM) $CD8^+$ T cells ($CD44^+CD62L^-$). **(I)** CD62L RB705 FMO and CD44 V450 FMO staining controls for $CD8^+$ T cells. **(J)** $CD4^+$ T cells were further characterized in a similar manner. EM $CD4^+$ T cells were further gated to identify T follicular helper (Tfh) cells ($CXCR5^+$PD-1^+). **(K)** CD62L RB705 FMO, CD44 V450 FMO, and CXCR5 PE/Dazzle 594 FMO staining controls for $CD4^+$ T cells. Data shown are representative of two mice (*n* = 2).

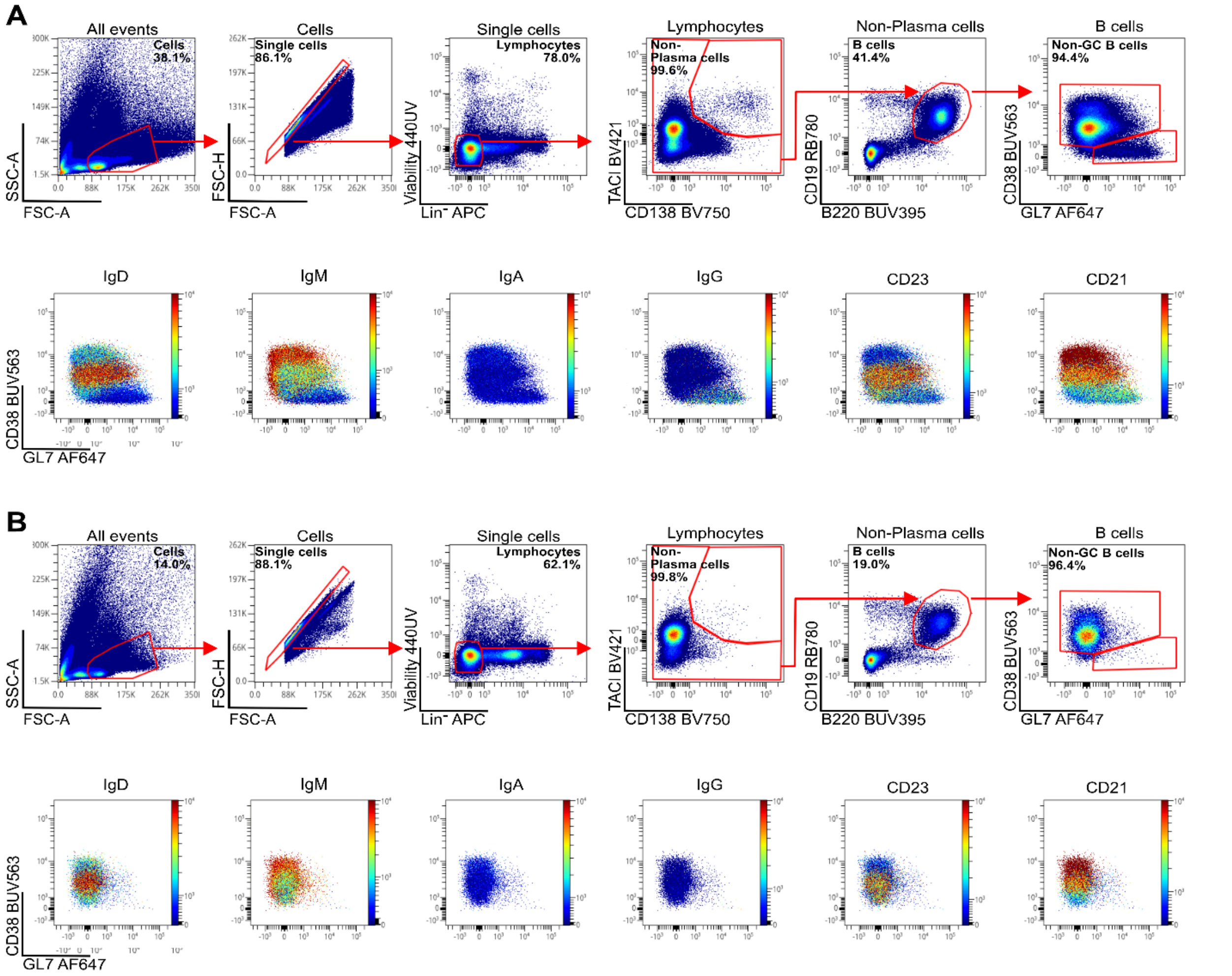

Supplementary Figure S7: Gating strategy and phenotypic characterization of CD38$^+$ non-GC B cell subsets in the spleen of immunized and non-immunized mice. (A) and (B) show representative data from an immunized **(A)** and a non-immunized **(B)** mouse. Gating strategy for viable lymphocytes following the exclusion of cellular debris, doublets, dead cells, and lineage-negative (Lin$^-$) cells (TER$^-$119$^+$, Ly-6G/Ly-6C$^+$ [Gr-1$^+$], F4/80$^+$, CD11c$^+$, NK-1.1$^+$). Plasma cells (PCs) and plasmablasts (PBs; TACI^{int}CD138+) were excluded from B cells (CD19$^+$B220$^+$). Non-germinal center (GC) B cells were defined by their expression of CD38 and the absence of the GL7 marker. Within the CD38$^+$ population the expression of several markers was analyzed to distinguish between CD23^{high}CD21$^+$ naive follicular, CD23^{low}CD21^{high} marginal zone, and IgG$^+$ class-switched B cells further on. Continuous expression overlays of the B cell gate are shown. Marker expression levels are visualized by color gradient. Data shown are representative of three mice (n = 3).

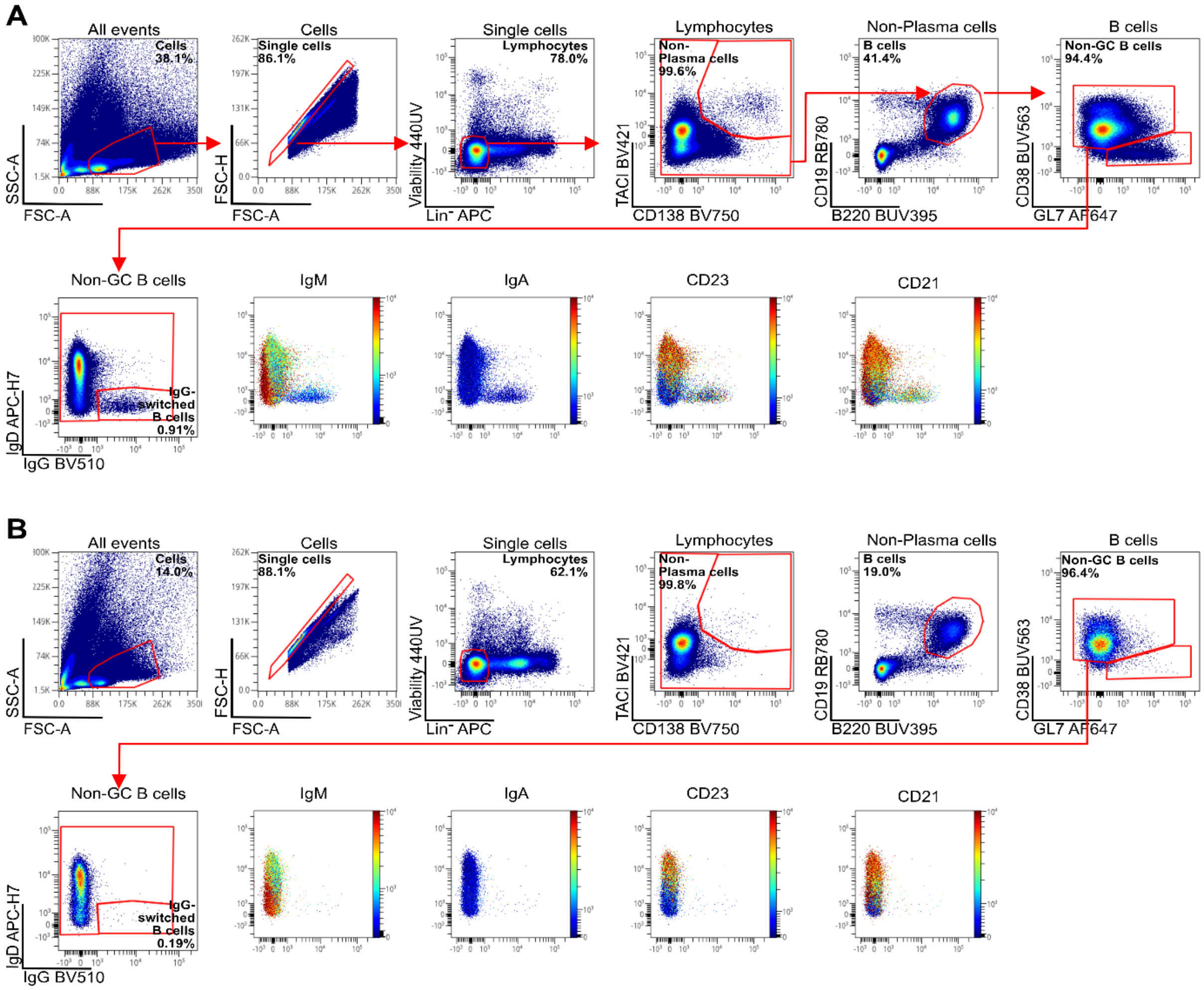

Supplementary Figure S8: Gating strategy and phenotypic characterization of IgG$^+$ class-switched B cells in the spleen of immunized and non-immunized mice. (A) and (B) show representative data from an immunized **(A)** and a non-immunized **(B)** mouse. Gating strategy for viable lymphocytes following the exclusion of cellular debris, doublets, dead cells, and lineage-negative (Lin$^-$) cells (TER$^-$119$^+$, Ly-6G/Ly-6C$^+$ [Gr-1$^+$], F4/80$^+$, CD11c$^+$, NK-1.1$^+$). Plasma cells (PCs) and plasmablasts (PBs; TACI^{int}CD138+) were excluded from B cells (CD19$^+$B220$^+$). Non-germinal center (GC) B cells were defined by their expression of CD38 and the absence of the GL7 marker. The CD38$^+$ population was further gated based on IgD and IgG expression to identify and characterize IgG$^+$ class-switched B cells. Continuous expression overlays of the non-GC B cell gate are shown. Marker expression levels are visualized as a color gradient. Data shown are representative of three mice (*n* = 3).

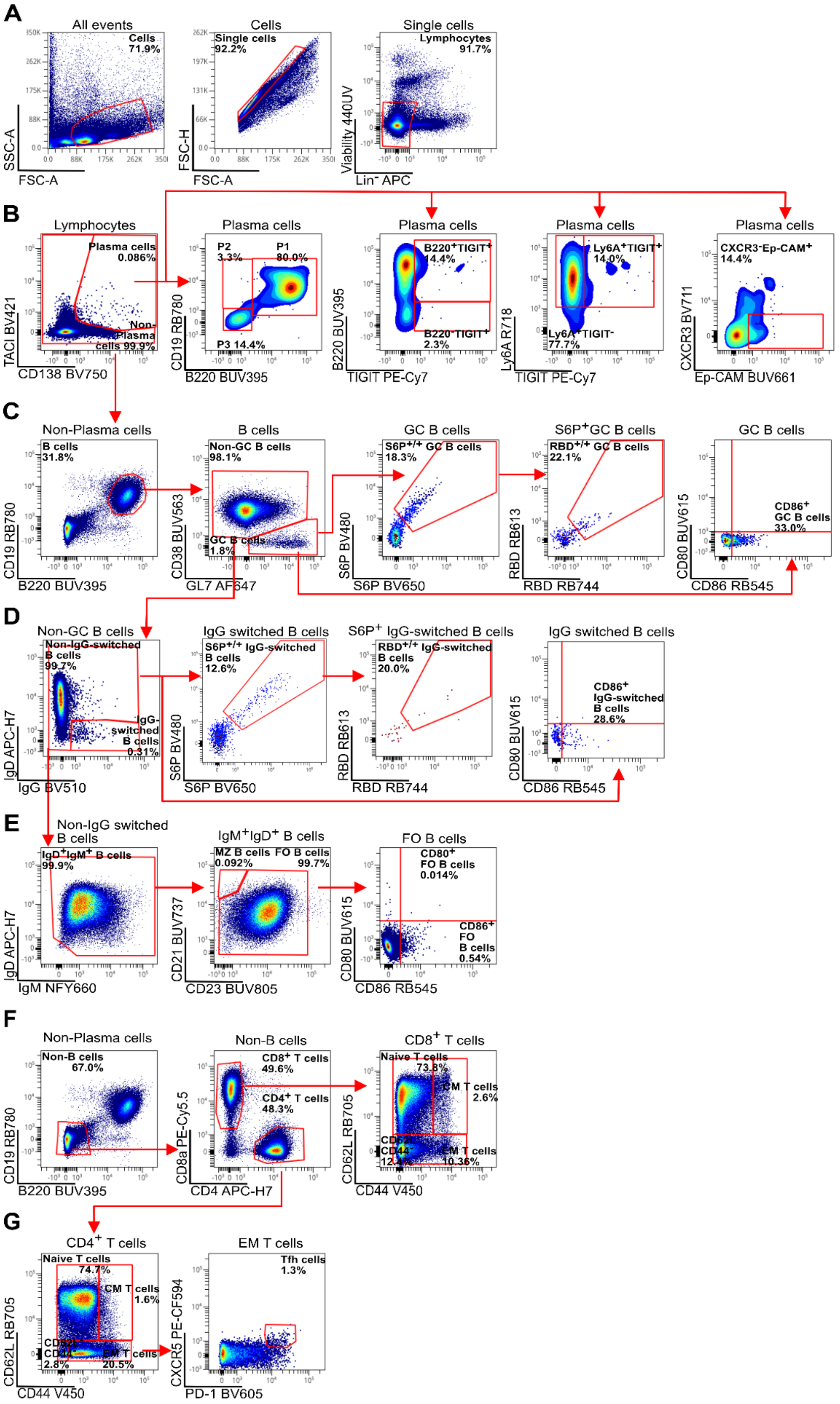
A
All events
Cells 71.9%
SSC-A
FSC-A
Cells
Single cells 92.2%
FSC-H
FSC-A
Single cells
Lymphocytes 91.7%
Viability 440UV
Lin- APC
B
Lymphocytes
Plasma cells 0.086%
Non-Plasma cells 99.9%
TACI BV421
CD138 BV750
Plasma cells
P2 3.3%
P1 80.0%
P3 14.4%
CD19 RB780
B220 BUV395
Plasma cells
B220+TIGIT+ 14.4%
B220-TIGIT+ 2.3%
B220 BUV395
TIGIT PE-Cy7
Plasma cells
Ly6A+TIGIT+ 14.0%
Ly6A+TIGIT- 77.7%
Ly6A R718
TIGIT PE-Cy7
Plasma cells
CXCR3-Ep-CAM+ 14.4%
CXCR3 BV711
Ep-CAM BUV661
C
Non-Plasma cells
B cells 31.8%
CD19 RB780
B220 BUV395
B cells
Non-GC B cells 98.1%
GC B cells 1.8%
CD38 BUV563
GL7 AF647
GC B cells
S6P+/+ GC B cells 18.3%
S6P BV480
S6P BV650
S6P+GC B cells
RBD+/+ GC B cells 22.1%
RBD RB613
RBD RB744
GC B cells
CD86+ GC B cells 33.0%
CD80 BUV615
CD86 RB545
D
Non-GC B cells
Non-IgG-switched B cells 99.7%
IgG-switched B cells 0.31%
IgD APC-H7
IgG BV510
IgG switched B cells
S6P+/+ IgG-switched B cells 12.6%
S6P BV480
S6P BV650
S6P+ IgG-switched B cells
RBD+/+ IgG-switched B cells 20.0%
RBD RB613
RBD RB744
IgG switched B cells
CD86+ IgG-switched B cells 28.6%
CD80 BUV615
CD86 RB545
E
Non-IgG switched B cells
IgD+IgM+ B cells 99.9%
IgD APC-H7
IgM NFY660
IgM+IgD+ B cells
MZ B cells 0.092%
FO B cells 99.7%
CD21 BUV737
CD23 BUV805
FO B cells
CD80+ FO B cells 0.014%
CD86+ FO B cells 0.54%
CD80 BUV615
CD86 RB545
F
Non-Plasma cells
Non-B cells 67.0%
CD19 RB780
B220 BUV395
Non-B cells
CD8+ T cells 49.6%
CD4+ T cells 48.3%
CD8a PE-Cy5.5
CD4 APC-H7
CD8+ T cells
Naive T cells 73.8%
CM T cells 2.6%
CD62L- CD44- 12.4%
EM T cells 10.36%
CD62L RB705
CD44 V450
G
CD4+ T cells
Naive T cells 74.7%
CM T cells 1.6%
CD62L- CD44- 2.8%
EM T cells 20.5%
CD62L RB705
CD44 V450
EM T cells
Tfh cells 1.3%
CXCR5 PE-CF594
PD-1 BV605

Supplementary Figure S9: Gating strategy for murine lymph node samples from an immunized mouse. (A) Gating strategy for viable lymphocytes following the exclusion of cellular debris, doublets, dead cells, and lineage-negative (Lin^{-}) cells ($TER^{-}119^{+}$, Ly-6G/Ly-$6C^{+}$ [Gr-1^{+}], F4/80^{+}, $CD11c^{+}$, NK-1.1^{+}). **(B)** Plasma cells (PCs) and plasmablasts (PBs; $TACI^{int}CD138+$) were further subdivided into three subsets: P1 – plasma cell precursors ($B220^{int}CD19^{int}$), P2 – early plasma cells ($B220^{lo}CD19^{int}$), and P3 – mature plasma cells ($B220^{low}CD19^{low}$). PCs expressing Ep-$CAM^{high}CXCR3^{-}$ or $Ly6A^{high}TIGIT^{-}$ phenotypes were identified. **(C)** Germinal center (GC) B cells ($CD38^{-}$ $GL7^{+}$) were investigated for antigen reactivity ($S6P^{+/+}$, $RBD^{+/+}$) as well as their activation status ($CD86^{+}$). **(D)** IgG^{+} class-switched B cells ($CD38^{+}GL7^{-}IgG^{+}$) showed antigen-reactivity ($S6P^{+/+}$, $RBD^{+/+}$) and expressed the activation marker CD86. **(E)** Naive follicular ($CD23^{high}CD21^{+}$) B cells were defined and investigated for their activation status ($CD80^{+}$). In contrast to the spleen, marginal zone ($CD23^{low}CD21^{high}$) B cells are not present in lymph nodes**. (F)** $CD8^{+}$ T cells were divided into naive $CD8^{+}$ T cells ($CD44^{-}CD62L^{+}$), central memory (CM) $CD8^{+}$ T cells ($CD44^{+}CD62L^{+}$), and effector memory (EM) $CD8^{+}$ T cells ($CD44^{+}CD62L^{-}$). **(G)** $CD4^{+}$ T cells were further characterized in a similar manner. EM $CD4^{+}$ T cells were further gated to identify T follicular helper (Tfh) cells ($CXCR5^{+}PD\text{-}1^{+}$). Data shown are representative of three mice (*n* = 3).

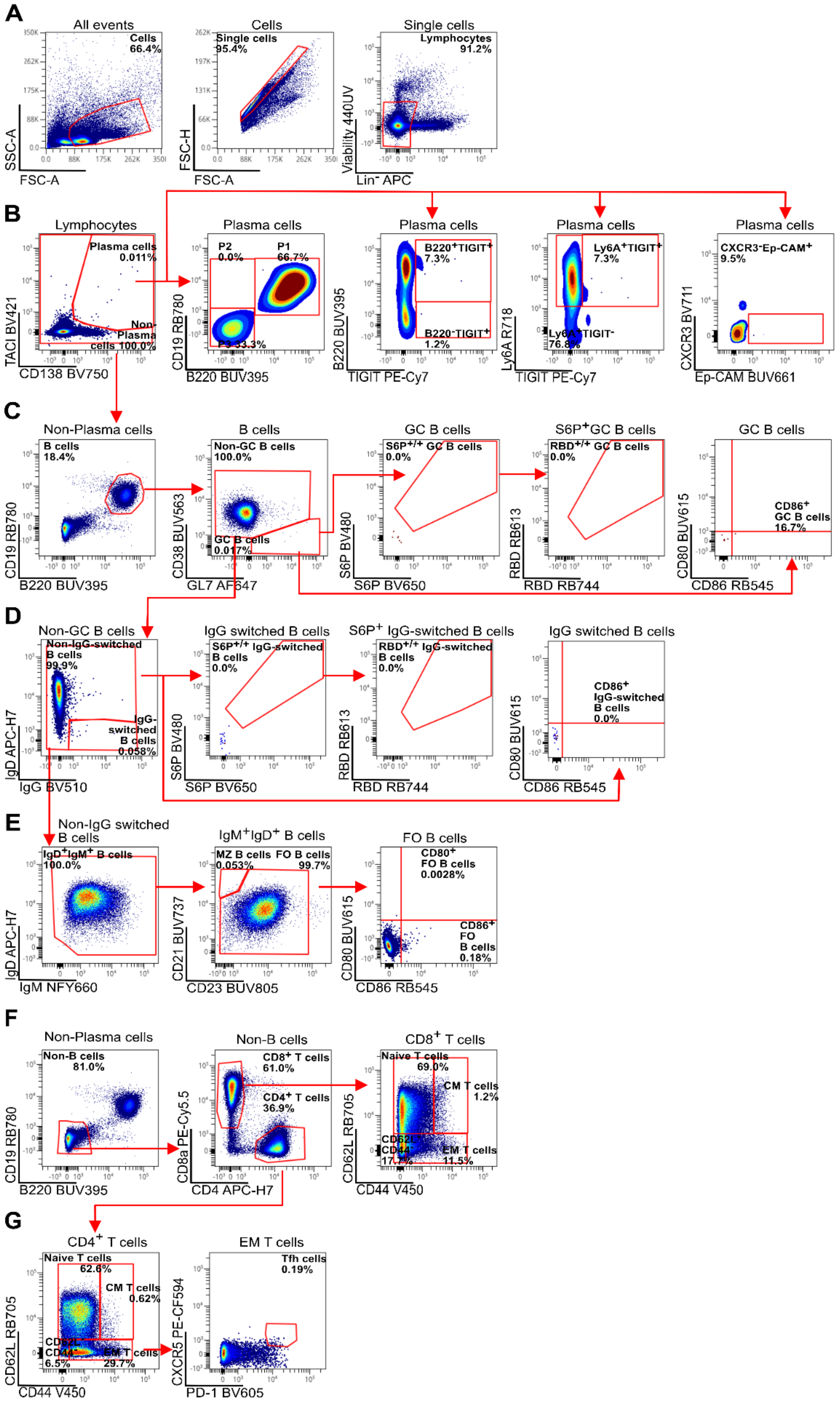

A
All events
Cells 66.4%
SSC-A
FSC-A
Cells
Single cells 95.4%
FSC-H
FSC-A
Single cells
Lymphocytes 91.2%
Viability 440UV
Lin⁻ APC
B
Lymphocytes
Plasma cells 0.011%
Non-Plasma cells 100.0%
TACI BV421
CD138 BV750
Plasma cells
P2 0.0%
P1 66.7%
P3 33.3%
CD19 RB780
B220 BUV395
Plasma cells
B220⁺TIGIT⁺ 7.3%
B220⁻TIGIT⁺ 1.2%
B220 BUV395
TIGIT PE-Cy7
Plasma cells
Ly6A⁺TIGIT⁺ 7.3%
Ly6A⁺TIGIT⁻ 76.8%
Ly6A R718
TIGIT PE-Cy7
Plasma cells
CXCR3⁻Ep-CAM⁺ 9.5%
CXCR3 BV711
Ep-CAM BUV661
C
Non-Plasma cells
B cells 18.4%
CD19 RB780
B220 BUV395
B cells
Non-GC B cells 100.0%
GC B cells 0.017%
CD38 BUV563
GL7 AF647
GC B cells
S6P⁺/⁺ GC B cells 0.0%
S6P BV480
S6P BV650
S6P⁺GC B cells
RBD⁺/⁺ GC B cells 0.0%
RBD RB613
RBD RB744
GC B cells
CD86⁺ GC B cells 16.7%
CD80 BUV615
CD86 RB545
D
Non-GC B cells
Non-IgG-switched B cells 99.9%
IgG-switched B cells 0.058%
IgD APC-H7
IgG BV510
IgG switched B cells
S6P⁺/⁺ IgG-switched B cells 0.0%
S6P BV480
S6P BV650
S6P⁺ IgG-switched B cells
RBD⁺/⁺ IgG-switched B cells 0.0%
RBD RB613
RBD RB744
IgG switched B cells
CD86⁺ IgG-switched B cells 0.0%
CD80 BUV615
CD86 RB545
E
Non-IgG switched B cells
IgD⁺IgM⁺ B cells 100.0%
IgD APC-H7
IgM NFY660
IgM⁺IgD⁺ B cells
MZ B cells 0.053%
FO B cells 99.7%
CD21 BUV737
CD23 BUV805
FO B cells
CD80⁺ FO B cells 0.0028%
CD86⁺ FO B cells 0.18%
CD80 BUV615
CD86 RB545
F
Non-Plasma cells
Non-B cells 81.0%
CD19 RB780
B220 BUV395
Non-B cells
CD8⁺ T cells 61.0%
CD4⁺ T cells 36.9%
CD8a PE-Cy5.5
CD4 APC-H7
CD8⁺ T cells
Naive T cells 69.0%
CM T cells 1.2%
CD62L⁻CD44⁻ 17.7%
EM T cells 11.5%
CD62L RB705
CD44 V450
G
CD4⁺ T cells
Naive T cells 62.6%
CM T cells 0.62%
CD62L⁻CD44⁻ 6.5%
EM T cells 29.7%
CD62L RB705
CD44 V450
EM T cells
Tfh cells 0.19%
CXCR5 PE-CF594
PD-1 BV605

Supplementary Figure S10: Gating strategy for murine lymph node samples from a non-immunized mouse. (A) Gating strategy for viable lymphocytes following the exclusion of cellular debris, doublets, dead cells, and lineage-negative (Lin^-) cells (TER^-119^+, Ly-6G/Ly-$6C^+$ [Gr-1^+], F4/80^+, $CD11c^+$, NK-1.1^+). **(B)** Plasma cells (PCs) and plasmablasts (PBs; $TACI^{int}CD138+$) were further subdivided into three subsets: P1 – plasma cell precursors ($B220^{int}CD19^{int}$), P2 – early plasma cells ($B220^{lo}CD19^{int}$), and P3 – mature plasma cells ($B220^{low}CD19^{low}$). PCs expressing Ep-$CAM^{high}CXCR3^-$ or $Ly6A^{high}TIGIT^-$ phenotypes were identified. **(C)** Germinal center (GC) B cells ($CD38^-$ $GL7^+$) were investigated for antigen reactivity ($S6P^{+/+}$, $RBD^{+/+}$) as well as their activation status ($CD86^+$). **(D)** IgG^+ class-switched B cells ($CD38^+GL7^-IgG^+$) showed no antigen-reactivity ($S6P^{+/+}$, $RBD^{+/+}$) and did not express the activation marker CD86. **(E)** Naive follicular ($CD23^{high}CD21^+$) B cells were defined and investigated for their activation status ($CD80^+$). In contrast to the spleen, marginal zone ($CD23^{low}CD21^{high}$) B cells are not present in lymph nodes**. (F)** $CD8^+$ T cells were divided into naive $CD8^+$ T cells ($CD44^-CD62L^+$), central memory (CM) $CD8^+$ T cells ($CD44^+CD62L^+$), and effector memory (EM) $CD8^+$ T cells ($CD44^+CD62L^-$). **(G)** $CD4^+$ T cells were further characterized in a similar manner. EM $CD4^+$ T cells were further gated to identify T follicular helper (Tfh) cells ($CXCR5^+PD$-1^+). Data shown are representative of two mice (*n* = 2).

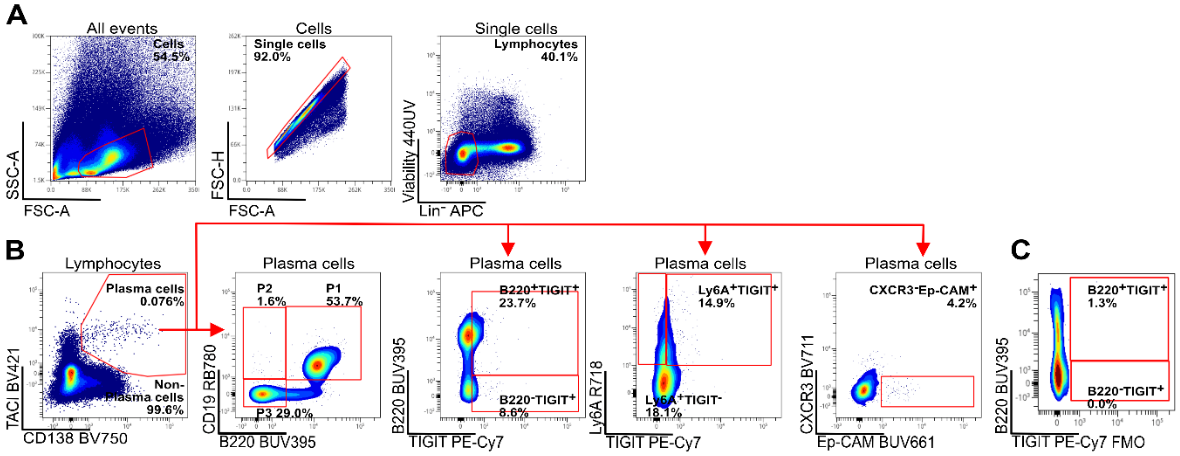

Supplementary Figure S11: Gating strategy for murine bone marrow plasma cell subsets from a non-immunized mouse. **(A)** Gating strategy for viable lymphocytes following the exclusion of cellular debris, doublets, dead cells, and lineage-negative (Lin⁻) cells (TER⁻119⁺, Ly-6G/Ly-6C⁺ [Gr-1⁺], F4/80⁺, CD11c⁺, NK-1.1⁺). **(B)** Plasma cells (PCs) and plasmablasts (PBs; TACI^{int}CD138+) were further subdivided into three subsets: P1 – plasma cell precursors (B220^{int}CD19^{int}), P2 – early plasma cells (B220^{lo}CD19^{int}), and P3 – mature plasma cells (B220^{low}CD19^{low}). PCs expressing Ep-CAM^{high}CXCR3^{-} or Ly6A^{high}TIGIT^{-} phenotypes were identified. **(C)** TIGIT PE-Cy7 FMO staining control. Data shown are representative of two mice (*n* = 2).

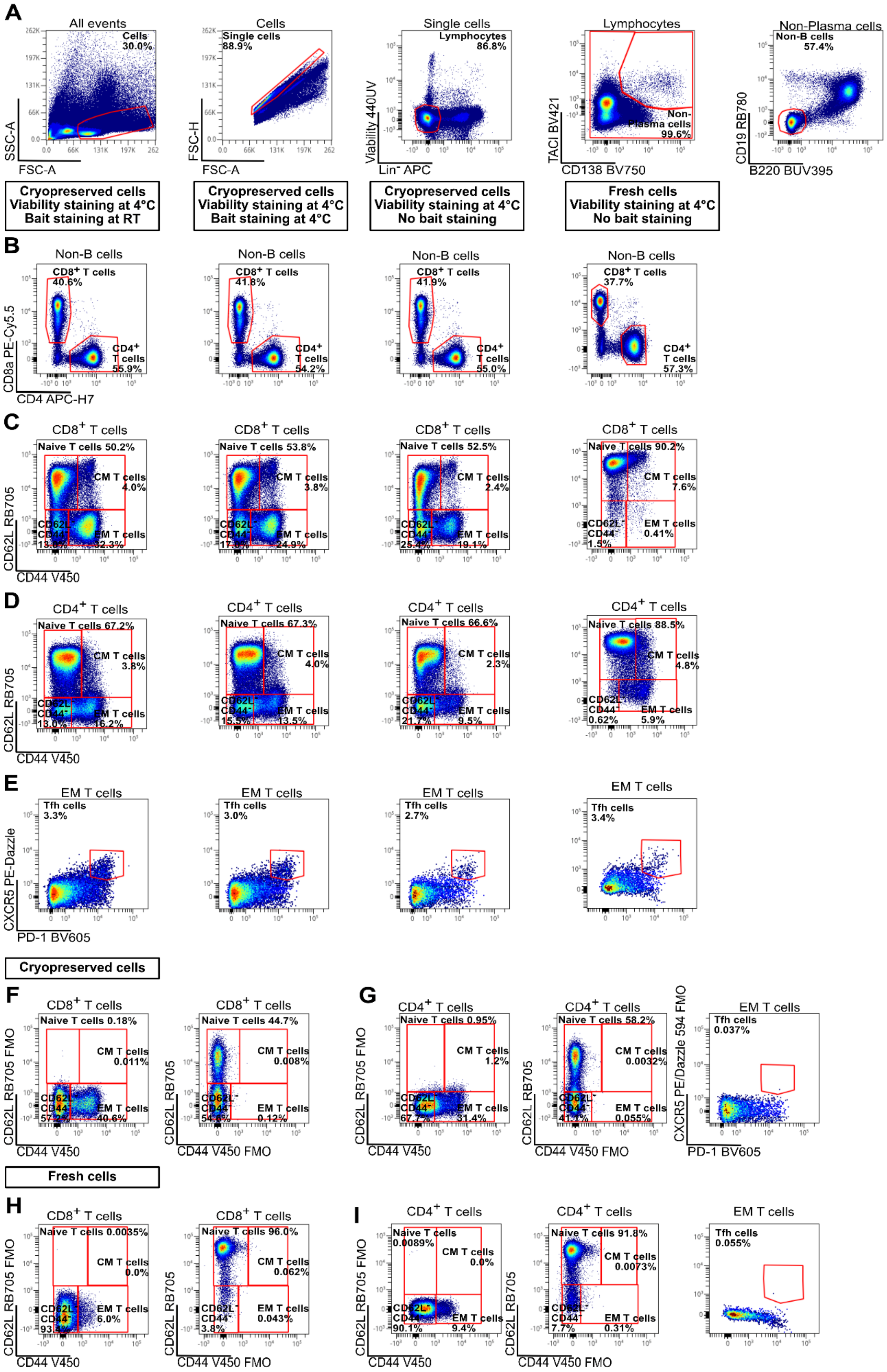

A
All events
Cells 30.0%
SSC-A
FSC-A
Cells
Single cells 88.9%
FSC-H
FSC-A
Single cells
Lymphocytes 86.8%
Viability 440UV
Lin⁻ APC
Lymphocytes
Non-Plasma cells 99.6%
TACI BV421
CD138 BV750
Non-Plasma cells
Non-B cells 57.4%
CD19 RB780
B220 BUV395
Cryopreserved cells Viability staining at 4°C Bait staining at RT
Cryopreserved cells Viability staining at 4°C Bait staining at 4°C
Cryopreserved cells Viability staining at 4°C No bait staining
Fresh cells Viability staining at 4°C No bait staining
B
Non-B cells
CD8⁺ T cells 40.6%
CD4⁺ T cells 55.9%
CD8⁺ T cells 41.8%
CD4⁺ T cells 54.2%
CD8⁺ T cells 41.9%
CD4⁺ T cells 55.0%
CD8⁺ T cells 37.7%
CD4⁺ T cells 57.3%
CD8a PE-Cy5.5
CD4 APC-H7
C
CD8⁺ T cells
Naive T cells 50.2%
CM T cells 4.0%
CD62L⁻ CD44⁻ 13.6%
EM T cells 32.3%
Naive T cells 53.8%
CM T cells 3.8%
CD62L⁻ CD44⁻ 17.5%
EM T cells 24.9%
Naive T cells 52.5%
CM T cells 2.4%
CD62L⁻ CD44⁻ 25.4%
EM T cells 19.1%
Naive T cells 90.2%
CM T cells 7.6%
CD62L⁻ CD44⁻ 1.5%
EM T cells 0.41%
CD62L RB705
CD44 V450
D
CD4⁺ T cells
Naive T cells 67.2%
CM T cells 3.8%
CD62L⁻ CD44⁻ 13.0%
EM T cells 16.2%
Naive T cells 67.3%
CM T cells 4.0%
CD62L⁻ CD44⁻ 15.5%
EM T cells 13.5%
Naive T cells 66.6%
CM T cells 2.3%
CD62L⁻ CD44⁻ 21.7%
EM T cells 9.5%
Naive T cells 88.5%
CM T cells 4.8%
CD62L⁻ CD44⁻ 0.62%
EM T cells 5.9%
CD62L RB705
CD44 V450
E
EM T cells
Tfh cells 3.3%
Tfh cells 3.0%
Tfh cells 2.7%
Tfh cells 3.4%
CXCR5 PE-Dazzle
PD-1 BV605
Cryopreserved cells
F
CD8⁺ T cells
Naive T cells 0.18%
CM T cells 0.011%
CD62L⁻ CD44⁻ 57.2%
EM T cells 40.6%
CD62L RB705 FMO
CD44 V450
Naive T cells 44.7%
CM T cells 0.008%
CD62L⁻ CD44⁻ 54.6%
EM T cells 0.12%
CD62L RB705
CD44 V450 FMO
G
CD4⁺ T cells
Naive T cells 0.95%
CM T cells 1.2%
CD62L⁻ CD44⁻ 67.7%
EM T cells 31.4%
CD62L RB705 FMO
CD44 V450
Naive T cells 58.2%
CM T cells 0.0032%
CD62L⁻ CD44⁻ 41.7%
EM T cells 0.055%
CD62L RB705
CD44 V450 FMO
EM T cells
Tfh cells 0.037%
CXCR5 PE/Dazzle 594 FMO
PD-1 BV605
Fresh cells
H
CD8⁺ T cells
Naive T cells 0.0035%
CM T cells 0.0%
CD62L⁻ CD44⁻ 93.9%
EM T cells 6.0%
CD62L RB705 FMO
CD44 V450
Naive T cells 96.0%
CM T cells 0.062%
CD62L⁻ CD44⁻ 3.8%
EM T cells 0.043%
CD62L RB705
CD44 V450 FMO
I
CD4⁺ T cells
Naive T cells 0.0089%
CM T cells 0.0%
CD62L⁻ CD44⁻ 90.1%
EM T cells 9.4%
CD62L RB705 FMO
CD44 V450
Naive T cells 91.8%
CM T cells 0.0073%
CD62L⁻ CD44⁻ 7.7%
EM T cells 0.31%
CD62L RB705
CD44 V450 FMO
EM T cells
Tfh cells 0.055%

Supplementary Figure S12: T cell staining on cryopreserved and freshly prepared murine spleen samples. Cryopreserved cells were derived from an immunized mouse, and freshly prepared cells from a non-immunized mouse. Different conditions were tested, as indicated above the plots and described in the Materials and Methods section of the Supplementary Information. **(A)** Gating strategy for viable lymphocytes following the exclusion of cellular debris, doublets, dead cells, and lineage-negative (Lin^-) cells (TER^-119^+, Ly-6G/Ly-$6C^+$ [Gr-1^+], F4/80^+, $CD11c^+$, NK-1.1^+). Plasma cells (PCs) and plasmablasts (PBs; $TACI^{int}CD138+$) as well as B cells ($CD19^+B220^+$) were excluded. **(B)** $CD8^+$ T cells were gated and further subdivided into **(C)** naive $CD8^+$ T cells ($CD44^-CD62L^+$), central memory (CM) $CD8^+$ T cells ($CD44^+CD62L^+$), and effector memory (EM) $CD8^+$ T cells ($CD44^+CD62L^-$). **(D)** $CD4^+$ T cells were analyzed using the same gating strategy. **(E)** EM $CD4^+$ T cells were further gated to identify T follicular helper (Tfh) cells ($CXCR5^+$PD-1^+). **(F, G)** FMO controls for cryopreserved $CD8^+$ T cells (F) and $CD4^+$ T cells (G) are indicated in the axis labels. **(H, I)** FMO controls for freshly prepared $CD8^+$ T cells (H) and $CD4^+$ T cells (I) are indicated in the axis labels. Data shown are representative of two mice (n = 2).

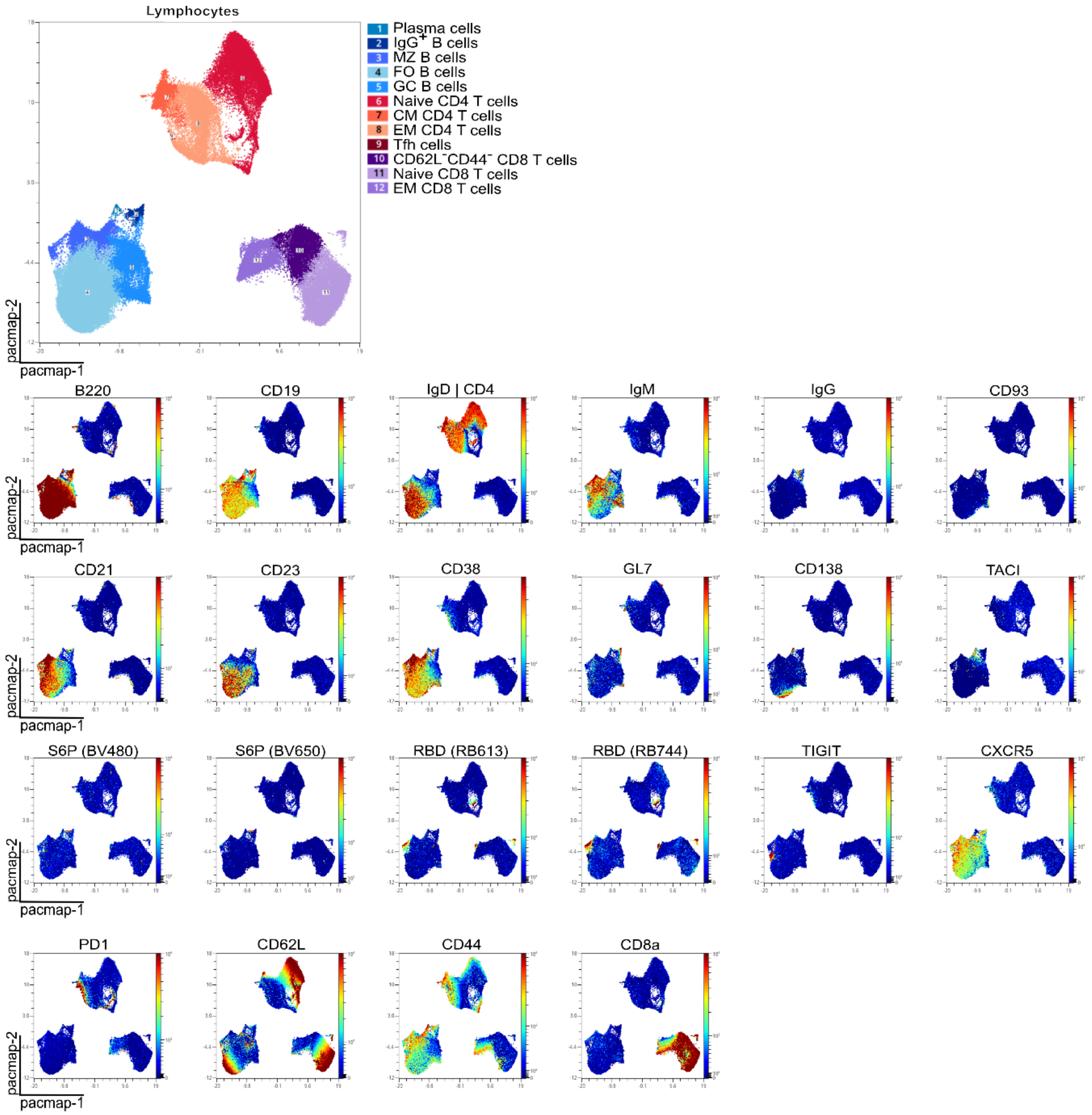

Supplementary Figure S13: Pairwise Controlled Manifold Approximation and Projection (PaCMAP) of lymphocytes collected from a spleen sample of an immunized mouse 7 days after secondary immunization. Prior to analysis, cellular debris, doublets, dead cells, and erythrocytes (TER-119$^+$), as well as monocytes and neutrophils (Ly6G/Ly-6C$^+$ (Gr-1$^+$)). macrophages (F4/80$^+$), dendritic cells (CD11c$^+$), and NK cells (NK-1.1$^+$) were excluded. Dimensionality reduction was performed using PaCMAP with the following settings: *k* nearest neighbors = 30, mid-near pair ratio = 0.5, further pair ratio = 2, iterations = 600, distance metric = Euclidean, initialization = random, and output dimensions = 2. FlowSOM clustering was subsequently applied on the marker expression matrix with metaclustering set to *k* = 16. Clusters are overlaid on the PaCMAP layout to visualize the phenotypic heterogeneity of B and T cell subsets. Continuous expression overlays of selected phenotypic markers used to annotate clusters. Marker expression levels are visualized as a color gradient on the PaCMAP projection. Data shown are representative of three mice (*n* = 3).

References

1. Verheijen M, Rane S, Pearson C, Yates AJ, Seddon B. Fate Mapping Quantifies the Dynamics of B Cell Development and Activation throughout Life. Cell Rep. 2020;33(7):108376.
2. Weisel NM, Joachim SM, Smita S, Callahan D, Elsner RA, Conter LJ, et al. Surface phenotypes of naive and memory B cells in mouse and human tissues. Nat Immunol. 2022;23(1):135-45.
3. Liu X, Yao J, Zhao Y, Wang J, Qi H. Heterogeneous plasma cells and long-lived subsets in response to immunization, autoantigen and microbiota. Nat Immunol. 2022;23(11):1564-76.
4. Liu Z, Gu Y, Shin A, Zhang S, Ginhoux F. Analysis of Myeloid Cells in Mouse Tissues with Flow Cytometry. STAR Protoc. 2020;1(1):100029.
5. Taylor MD, Brewer MR, Nedeljkovic-Kurepa A, Yang Y, Reddy KS, Abraham MN, et al. CD4 T Follicular Helper Cells Prevent Depletion of Follicular B Cells in Response to Cecal Ligation and Puncture. Front Immunol. 2020;11:1946.
6. Mincham KT, Young JD, Strickland DH. OMIP 076: High-dimensional immunophenotyping of murine T-cell, B-cell, and antibody secreting cell subsets. Cytometry A. 2021;99(9):888-92.
7. Pracht K, Meinzinger J, Daum P, Schulz SR, Reimer D, Hauke M, et al. A new staining protocol for detection of murine antibody-secreting plasma cell subsets by flow cytometry. Eur J Immunol. 2017;47(8):1389-92.

Supplementary Information | Tables

Supplementary Table S1: Antibody clones and fluorochromes

Target specificity	Alternative Name	Clone	Fluorochrome	Purpose
CD45R	B220	RA3-6B2	BUV395	Pan-B cell lineage
Live/Dead		N/A	Fixable Viability Stain 440UV	Viable cells
CD38		90	BUV563	B cell developmental/maturation stages
CD80		16-10A1	BUV615	B cell activation/antigen-presentation
CD326	Ep-CAM	G8.8	BUV661	Plasma cell differentiation
CD21		7G6	BUV737	Marginal zone B cells
CD23		B3B4	BUV805	Follicular B cells
CD267	TACI	8F10	BV421	B cell survival and class switching
CD44		IM7	V450	Activation and memory
S6P		N/A	BV480	Antigen-reactivity
IgG1		RMG1-1	BV510	Switching (Th2-associated isotype)
IgG2a/b		R2-40	BV510	Switching (Th1-associated isotype)
IgG3		R40-82	BV510	Switching (Complement-fixing isotype)
CD279	PD-1	29F.1A12	BV605	T cell exhaustion checkpoint
S6P		N/A	BV650	Antigen-reactivity
CD183	CXCR3	CXCR3-173	BV711	Long-lived plasma cell
CD138	Synd-1	281-2	BV750	Plasma cell differentiation
IgA		C10-3	FITC	Switching (mucosal/T-independent response)
CD86		PO3	RB545	B cell activation/antigen-presentation
RBD		N/A	RB613	Antigen-reactivity
CD62L		MEL-14	RB705	Naive/Central memory T cell
RBD		N/A	RB744	Antigen-reactivity
CD19		1D3	RB780	Pan-B cell lineage
CD93	AA4.1	AA4.1	RY586	Transitional B cells
CD185	CXCR5	L138D7	PE/Dazzle 594	Germinal center B cell and Tfh cell
IgM		II/41	NovaFluor™ Yellow 660	Naive/unswitched B cell
CD8a		53-6.7	PE-Cy5.5	Cytotoxic T cell lineage
TIGIT		GIGD7	PE-Cy7	Plasma cell differentiation

Target specificity	Alternative Name	Clone	Fluorochrome	Purpose
T- and B- Cell Activation Antigen	GL7	GL7	AF647	Germinal center B cell
NK-1.1		PK136	APC	Natural killer cell exclusion
TER-119		TER-119	APC	Erythroid lineage exclusion
F4/80		T45-2342	APC	Macrophage/Monocyte exclusion
Ly-6G/Ly-6C (Gr-1)		RB6-8C5	APC	Myeloid/Neutrophil exclusion
CD11c		HL3	APC	Dendritic cell exclusion
Lymphocyte antigen 6A-2/6E-1	Ly6A	D7	R718	Plasma cell differentiation
IgD		11-26c.2a	APC-H7	Naive B cell
CD4		GK1.5	APC-H7	Helper T cell lineage

Supplementary Table S2: Configuration of the full visible spectrum cytometer BD FACSymphony™ A5 SE with five lasers and 49 detectors.

Wavelength (nm)/laser	Laser power (mW)	Detector*	Longpass optical filter	Bandpass (nm) optical filter	Fluorochrome (Peak intensity)
**UV laser**					
355/UV	65	A	765LP	809/82	BUV805
		B	704LP	736/64	BUV737
		C	675LP	695/40	
		D	645LP	660/30	BUV661
		E	595LP	610/30	BUV615
		F	570LP	585/30	BUV563
		G	535LP	540/20	Autofluorescence measurement
		H	495LP	515/30	
		I	425LP	446/67	Fixable Viability Stain 440UV
		J	365LP	379/34	BUV395
**Violet laser**					
405/Violet	200	A	810LP	845/70	
		B	765LP	785/50	
		C	730LP	750/40	BV750
		D	690LP	710/40	BV711
		E	665LP	680/30	
		F	645LP	660/30	BV650
		G	605LP	615/25	
		H	585LP	595/30	BV605
		I	570LP	576/20	
		J	530LP	540/20	
		K	495LP	510/40	BV510
		L	465LP	470/15	BV480
		M	430LP	450/40	V450
		N	415LP	431/28	BV421
**Blue laser**					
488/Blue	150	A	770LP	810/79	RB780
		B	724LP	750/60	RB744
		C	685LP	710/50	RB705
		D	665LP	675/20	
		E	645LP	660/30	
		F	585LP	602/40	RB613
		G	570LP	576/20	RB545
		H	520LP	537/32	
		I	500LP	510/20	FITC
		J	-	488/10	SSC

Wavelength (nM)/Laser	Laser power (mW)	Detector	Longpass	Bandpass (nm)	Fluorochrome (Peak intensity)

Yellow Green laser					
561/Yellow-gree	150	A	800LP	825/49	
		B	750LP	780/60	PE-Cy7
		C	735LP	750/40	
		D	699LP	730/50	
		E	680LP	695/40	PeCy5.5
		F	665LP	670/20	
		G	645LP	660/30	NovaFluor™ Yellow 660
		H	595LP	602/40	PE/Dazzle 594
		I	570LP	585/30	RY586

Red laser					
640/Red	140	A	750LP	780/60	APC-H7
		B	720LP	730/50	R718
		C	699LP	710/25	
		D	680LP	680/30	APC
		E	665LP	675/20	AF647
		F	645LP	662/11	

Supplementary Table S3: Antibody list

Antigen	Fluorochrome	Clone	Manufacturer	Catalog #	Dilution
B220	BUV395	RA3-6B2	BD Biosciences	563793	1:100
Live/Dead	Fixable Viability Stain 440UV	N/A	BD Biosciences	566332	1:500
CD38	BUV563	90	BD Biosciences	741271	1:200
CD80	BUV615	16-10A1	BD Biosciences	751328	1:100
Ep-CAM	BUV661	G8.8	BD Biosciences	741592	1:100
CD21	BUV737	7G6	BD Biosciences	612810	1:1600
CD23	BUV805	B3B4	BD Biosciences	741922	1:200
TACI	BV421	8F10	BD Biosciences	742840	1:100
CD44	V450	IM7	BD Biosciences	560451	1:400
S6P / Streptavidin	BV480	N/A	Acrobiosystems/ BD Biosciences	SPN-C82E9 / 564876	-
IgG1	BV510	RMG1-1	BioLegend	406621	1:100
IgG2a/b	BV510	R2-40	BD Biosciences	744293	1:100
IgG3	BV510	R40-82	BD Biosciences	744134	1:100
PD-1	BV605	29F.1A12	BD Biosciences	568867	1:100
S6P / Streptavidin	BV650	N/A	Acrobiosystems/ BioLegend	SPN-C82E9 / 405232	-
CXCR3	BV711	CXCR3-173	BD Biosciences	740825	1:100
CD138	BV750	281-2	BD Biosciences	747070	1:400
IgA	FITC	C10-3	BD Biosciences	559354	1:200
CD86	RB545	PO3	BD Biosciences	756392	1:100
RBD / Streptavidin	RB613	N/A	Acrobiosystems/ BD Biosciences	SPD-C82E9 / 571111	-
CD62L	RB705	MEL-14	BD Biosciences	570282	1:1000
RBD / Streptavidin	RB744	N/A	Acrobiosystems/ BD Biosciences	SPD-C82E9 / 570516	-
CD19	RB780	1D3	BD Biosciences	755522	1:800
CD93	RY586	AA4.1	BD Biosciences	753131	1:100
CXCR5	PE/Dazzle 594	L138D7	BioLegend	145522	1:200
IgM	NovaFluor™ Yellow 660	II/41	BD Biosciences / Thermo Fisher Scientific	553435 / K06T04L007	1:100
CD8a	PE-Cy5.5	53-6.7	FisherScientific	35-0081-82	1:400
TIGIT	PE-Cy7	GIGD7	Invitrogen	25-9501-82	1:100
GL7	AF647	GL7	BD Biosciences	561529	1:100
NK-1.1	APC	PK136	BD Biosciences	550627	1:100
TER-119	APC	TER-119	BD Biosciences	557909	1:100
F4/80	APC	T45-2342	BD Biosciences	566787	1:200
Ly-6G/Ly-6C (Gr-1)	APC	RB6-8C5	BD Biosciences	561083	1:100
CD11c	APC	HL3	BD Biosciences	550261	1:100

Antigen	Fluorochrome	Clone	Manufacturer	Catalog #	Dilution
Ly6A	R718	D7	BD Biosciences	567733	1:400
IgD	APC-H7	11-26c.2a	BD Biosciences	565348	1:200
CD4	APC-H7	GK1.5	BioLegend	560181	1:400

Supplementary Table S4: List of single stains used for unmixing of fluorochromes.

Fluorochrome	Cells/Beads*	Reference Control
BUV395	Beads	B220
Fixable Viability Stain 440UV	Cells	Live/Dead
BUV563	Beads	CD38
BUV615	Beads	CD80
BUV661	Beads	Ep-CAM
BUV737	Beads	CD21
BUV805	Beads	CD23
BV421	Beads	TACI
V450	Beads	CD44
BV480	Cells	S6P / Streptavidin substitute
BV510	Beads	IgG1
BV510	Beads	IgG2a/b
BV510	Beads	IgG3
BV605	Beads	PD-1
BV650	Cells	S6P / Streptavidin substitute
BV711	Beads	CXCR3
BV750	Beads	CD138
FITC	Beads	IgA
RB545	Beads	CD86
RB613	Cells	RBD / Streptavidin substitute
RB705	Cells	CD62L
RB744	Cells	RBD / Streptavidin substitute
RB780	Beads	CD19
RY586	Beads	CD93
PE/Dazzle 594	Beads	CXCR5
NovaFluor™ Yellow 660	Cells	IgM
PE-Cy5.5	Cells	CD8a
PE-Cy7	Beads	TIGIT
AF647	Beads	GL7
APC	Beads	NK-1.1
APC	Beads	TER-119
APC	Beads	F4/80
APC	Beads	Ly-6G/Ly-6C (Gr-1)
APC	Beads	CD11c
R718	Cells	Ly6A
APC-H7	Beads	IgD
APC-H7	Beads	CD4

* Single stains of each antibody were prepared using either cells or beads (splenocytes, bone marrow-derived lymphocytes, or UltraComp eBeads™). Beads were selected as single-color reference controls when they produced spectral unmixing comparable to that of cell-based controls. For antigen baits, a surrogate control using antibodies targeting abundantly expressed markers, conjugated to the same fluorochrome and obtained from the same manufacturer, provided satisfactory results.

Supplementary Table S5: Panel design and optimization.

Bandpass (nm)	Iteration 1	Iteration 2	Iteration 3	Iteration 4	Iteration 5
**809/82**	CD23 (B3B4)	CD23 (B3B4)	CD23 (B3B4)	CD23 (B3B4)	CD23 (B3B4)
**736/64**	CD21 (7G6)	CD21 (7G6)	CD21 (7G6)	CD21 (7G6)	CD21 (7G6)
**695/40**					
**660/30**	Ep-CAM (G8.8)	Ep-CAM (G8.8)	Ep-CAM (G8.8)	Ep-CAM (G8.8)	Ep-CAM (G8.8)
**610/30**	CD80 (16-10A1)	CD80 (16-10A1)	CD80 (16-10A1)	CD80 (16-10A1)	CD80 (16-10A1)
**585/30**	CD95 (Jo2)	CD38 (90)	CD38 (90)	CD38 (90)	CD38 (90)
**540/20**	Autofluorescence measurement	Autofluorescence measurement	Autofluorescence measurement	Autofluorescence measurement	Autofluorescence measurement
**515/30**	S6P				
**446/67**	Fixable Viability Stain 440UV	Fixable Viability Stain 440UV	Fixable Viability Stain 440UV	Fixable Viability Stain 440UV	Fixable Viability Stain 440UV
**379/34**	B220 (RA3-6B2) CD4 (GK1.5)	B220 (RA3-6B2)	B220 (RA3-6B2)	B220 (RA3-6B2)	B220 (RA3-6B2)
**845/70**	IgA (C10-1)	IgA (C10-1)	IgA (C10-1)		
**785/50**					
**750/40**	CD138 (281-2)	CD138 (281-2)	CD138 (281-2)	CD138 (281-2)	CD138 (281-2)
**710/40**	CXCR3 (CXCR3-173)	CXCR3 (CXCR3-173)	CXCR3 (CXCR3-173)	CXCR3 (CXCR3-173)	CXCR3 (CXCR3-173)
**680/30**					
**660/30**	PD-1 (29F.1A12)	S6P	S6P	S6P	S6P
**615/25**					
**595/30**	CD205 (NLDC-145)	PD-1 (29F.1A12)	PD-1 (29F.1A12)	PD-1 (29F.1A12)	PD-1 (29F.1A12)
**576/20**					
**540/20**					
**510/40**	IgM (II/41)	IgG1 (29F.1A12) IgG2a/b (R2-40) IgG3 (R40-82)	IgG1 (29F.1A12) IgG2a/b (R2-40) IgG3 (R40-82)	IgG1 (29F.1A12) IgG2a/b (R2-40) IgG3 (R40-82)	IgG1 (29F.1A12) IgG2a/b (R2-40) IgG3 (R40-82)
**470/15**	S6P	S6P	S6P	S6P	S6P
**450/40**	CD44 (IM7)	CD44 (IM7)	CD44 (IM7)	CD44 (IM7)	CD44 (IM7)
**431/28**	TACI (8F10)	TACI (8F10)	TACI (8F10)	TACI (8F10)	TACI (8F10)
**810/79**	CD19 (1D3)	CD19 (1D3)	CD19 (1D3)	CD19 (1D3)	CD19 (1D3)
**750/60**			RBD	RBD	RBD
**710/50**	PD-L1 (MIH5)			CD62L (MEL-14)	CD62L (MEL-14)
**675/20**					
**660/30**					
**602/40**			RBD	RBD	RBD
**576/20**	CD86 (PO3)	CD86 (PO3)	CD86 (PO3)	CD86 (PO3)	CD86 (PO3)
**537/32**					
**510/20**	IgD (11-26c.2a)	IgD (11-26c.2a) CD4 (GK1.5)	IgD (11-26c.2a) CD4 (GK1.5)	IgA (C10-3)	IgA (C10-3)
**488/10**	SSC	SSC	SSC	SSC	SSC
**825/49**					
**780/60**	TIGIT (1G9)	TIGIT (1G9)	TIGIT (1G9)	TIGIT (1G9)	TIGIT (GIGD7)
**750/40**					
**730/50**					
**695/40**	CD8a (53-6.7)	CD8a (53-6.7)	CD8a (53-6.7)	CD8a (53-6.7)	CD8a (53-6.7)
**670/20**					
**660/30**	IgG (polyclonal)	IgM (II/41)	IgM (II/41)	IgM (II/41)	IgM (II/41)
**602/40**	CXCR5 (2G8)	CXCR5 (2G8)	CXCR5 (2G8)	CXCR5 (2G8)	CXCR5 (L138D7)
**585/30**	CD93 (AA4.1)	CD93 (AA4.1)	CD93 (AA4.1)	CD93 (AA4.1)	CD93 (AA4.1)

Bandpass (nm)	Iteration 1	Iteration 2	Iteration 3	Iteration 4	Iteration 5
**780/60**	CD62L (MEL-14)	CD62L (MEL-14)	CD62L (MEL-14)	IgD (11-26c.2a)<br>CD4 (GK1.5)	IgD (11-26c.2a)<br>CD4 (GK1.5)
**730/50**	Ly6A (D7)	Ly6A (D7)	Ly6A (D7)	Ly6A (D7)	Ly6A (D7)
**710/25**					
**680/30**	Lineage$^-$	Lineage$^-$	Lineage$^-$	Lineage$^-$	Lineage$^-$
**675/20**	GL7 (GL7)	GL7 (GL7)	GL7 (GL7)	GL7 (GL7)	GL7 (GL7)
**662/11**					

Supplementary Table S6: Reasons underlying panel optimization and iteration decisions

Specificity	Fluorochrome	Clone	Reason
CD95	BUV563	Jo2	Did not separate germinal center B cells well enough.
CD38	BUV563	90	Replacement for anti-CD95 antibody for better separation of germinal center B cells.
CD4	BUV395	GK1.5	Shared fluorochrome between anti-B220 and anti-CD4 antibodies is not compatible with the gating strategy (separation between T cells and B220^{dim} B cells not clear). Therefore, anti-CD4 antibody was switched to FITC.
PD-1	BV650	29F.1A12	Anti-PD-1 antibody was switched to BV605 to free BV650 for S6P bait staining.
CD205	BV605	NLDC-145	Did not separate memory B cells optimally.
IgM	FITC	II/41	Anti-IgM antibody was switched to NovaFluor™ Yellow 660 to free FITC for the only combination of anti-IgG antibodies that would exclusively stain surface IgG.
IgG	NovaFluor™ Yellow 660	polyclonal	This antibody did not exclusively stain IgG but showed high cross-reactivity with other surface immunoglobulins.
IgG1<br>IgG2a/b<br>IgG3	FITC	29F.1A12<br>R2-40<br>R40-82	This combination was the only one we found to reliably stain surface IgG without cross-reactivity to other immunoglobulins.
RBD	RB613<br>RB744	-	RBD was added as additional bait.
IgA	BV786	C10-1	This anti-IgA clone was removed because of interactions with the anti-IgG1, anti-IgG2a/b, and anti-IgG3 antibody clones in a mix.
IgA	FITC	C10-3	This anti-IgA clone showed less cross-reactivity with the anti-IgG1, anti-IgG2a/b, anti-IgG3, and anti-IgM antibody clones in the mix.
CD4	FITC	GK1.5	Anti-CD4 antibody was switched to APC-H7 to free FITC.
IgD	FITC	11-26c.2a	Anti-IgD antibody was switched to APC-H7 to free FITC.
CD62L	APC-Cy7	MEL-14	Anti-CD62L was switched to RB705 to free channel 780/60.
TIGIT	PE-Cy7	1G9	Anti-TIGIT clone 1G9 is a mouse IgG1, κ antibody that interacts with the anti-IgG1 antibody, leading to false-positive IgG populations.
TIGIT	PE-Cy7	GIGD7	Anti-TIGIT clone GIGD7 is a rat IgG2a, κ antibody that avoids the issues described above.
CXCR5	PE-CF594	2G8	Anti-CXCR5 antibody shows reliable staining of B cells but does not strongly bind T follicular helper cells.
CXCR5	PE/Dazzle 594	L138D7	Anti-CXCR5 antibody also shows reliable staining of T follicular helper cells.

Orange filling: newly included reagents

Green filling: reagents that were replaced

Blue filling: reagents for which the fluorochrome was changed
